# Phytochemical characterization and fungal screening of *Sonneratia apetala* fruit and products: Pectin and vitamin C extraction, amino acids and antioxidant activity

**DOI:** 10.1371/journal.pone.0352259

**Published:** 2026-06-26

**Authors:** Md. Ripaj Uddin, Muhammad Abdullah Al Mansur, Hayatullah, Debabrata Karmakar, Nisat Taslum Jhumur, Amena Kibria, Shyama Prosad Moulick, Abubakr M. Idris

**Affiliations:** 1 Institute of National Analytical Research and Service (INARS), BCSIR, Dhaka, Bangladesh; 2 Institute of Mining, Mineralogy and Metallurgy (IMMM), Bangladesh Council of Scientific and Industrial Research (BCSIR), Joypurhat, Bangladesh; 3 Institute of Technology Transfer and Innovation (ITTI), BCSIR, Dhaka, Bangladesh; 4 Institute of Food Science and Technology (IFST), BCSIR, Dhaka, Bangladesh; 5 Chemical Research Division, BCSIR Laboratories, BCSIR, Dhaka, Bangladesh; 6 Department of Chemistry, King Khalid University, College of Science, Abha, Saudi Arabia; 7 Research Center for Advanced Materials Science (RCAMS), King Khalid University, Abha, Saudi Arabia; Ataturk University: Ataturk Universitesi, TÜRKIYE

## Abstract

The increasing demand for sustainable bio-resources has spurred interest in underutilized species like *Sonneratia apetala*, a mangrove with traditionally consumed fruits. This study provides a comprehensive biochemical and microbiological profile of *S. apetala* fruit and its derived products (jam, jelly, and pickle), integrating nutritional valorization with safety assessment. Pectin was extracted via acid hydrolysis and ethanol precipitation, yielding 2% with high purity (99.9%), confirmed by FT-IR and NMR spectroscopy. Vitamin C was isolated with a 1% yield and 99.9% purity, verified by HPLC and NMR. Amino acid profiling revealed the raw fruit was rich in essential amino acids, notably histidine (26.6 mg/g). Processing significantly degraded most amino acids, though histidine showed remarkable stability (16.4–17.8 mg/g). Antioxidant analysis demonstrated exceptional activity; the ethyl acetate root extract exhibited a potent DPPH IC₅₀ of 0.74 µg/mL, surpassing many synthetic antioxidants, alongside high Total Phenolic and Flavonoid Contents (555.8 mg GAE/100g DW and 240.6 mg QE/100g DW, respectively). A strong correlation was observed between phenolic content and antioxidant capacity. Crucially, fungal screening showed no detectable growth (<10 CFU/mL) in all samples, indicating product safety and stability. In conclusion, *S. apetala* fruit is a promising source of high-quality pectin, vitamin C, and potent antioxidants, with processed products being microbiologically stable. This research positions *S. apetala* as a valuable, multi-purpose species for nutraceutical and food industrial applications, with recommendations for further investigation into specific root antioxidants and scaled-up pectin extraction.

## 1. Introduction

The rising world demand for sustainable and natural bio-resource has rekindled interest in under-exploited plants with promising nutritional and industrial applications [1]. *Sonneratia apetala* Buch.-Ham., a fast-growing tree of the mangroves occurring commonly along south and southeast Asian coastslines, is locally consumed by communities for whom its inclusion in mainstream food and nutraceutical discourse remains remarkably limited [2,3]. It is used for its edible fruits, which can be taken raw or as pickles [4] and beverages, and believed to be rich in bioactive compounds such as vitamin C, pectin, essential amino acids, and microbes including fungi [5,6]. Despite observational1fresh fruit usage, scientific investigation of the compositional and functional properties has been sparse [7]. Vitamin C (ascorbic acid) is an essential water-soluble antioxidant that participates in many physiological processes, such as immune defense, collagen formation and iron uptake [8,9]. Fruit is an excellent source of vitamin C, and preliminary phytochemical studies showed that *S. apetala* could serve as a valuable source of this nutrient [10].

Concomitantly, pectin: complex poly-saccharide mainly located in fruit cell walls is a significant gelling and stabilizing agent used in the food industry. It also has functional health properties as cholesterol-lowering and prebiotic effects [11,12]. Most commercial pectin are extracted from the peel of oranges and lemons [13]. In this respect, the use of pectin has broadened and other sources have appeared as a replacement for example fig seed [14], passion fruit peel [15,16], mango peel [17,18] and guava pulp [19]. Although citrus and apple continue to be the primary origins for commercial production of pectins [13,20], tropical/mangrove fruits such as *S. apetala* are showing potential as alternative sources on account of their high productivity and quality.

The amino acid profile of plant-derived foods is as important, specifically with regards to its essential amino acid (EAA) content; those that human body cannot endogenously produce. EAAs, including lysine, leucine, valine and tryptophan are important for protein synthesis, metabolic control and muscle maintenance [21,22]. Consequently, determining the essential amino acid (EAA) profile of *S. apetala* fruit may provide valuable insights into its nutritional value and its potential to address protein malnutrition in resource-limited regions. Further, the advent and location of fungi (beneficial and pathogenic) on or inside fruit may affect shelf life, safety, and fermentability. Mangrove wetlands support a distinct microbiota with novel metabolic traits [23,24], however the fungal composition of *S. apetala* fruit has not been studied systematically. This is one of the very few studies to attain a complete biochemical and microbiological anatomy of *S. apetala* fruit and its product. Although some nutritional potential of mangrove species has been previously reported [23], this combination of nutritional profiling (vitamin C, pectin and amino acid) with fungal identification in both raw and processed products is a novel contribution. This type of information is vital to advocate the nutritional value of an underused fruit, as well as product safety and quality for commercialization. DPPH radical scavenging assay was employed to assess the in vitro antioxidant activity of the extracts. The study is focused on laboratory-scale analysis of the fruits for selected coastal mangrove ecosystems with potential implications for industrial scaling and further product development. These results will provide scientific supports for the development and utilization of *S. apetala* as a nutrition healthy fruit and industrial raw-material plant. Moreover, the detection of related fungi gives insight into safety aspects and can provide hints about strains with potential biotechnological importance.

This study aims to assess the biochemical and microbiological characteristics of *S. apetala* fruit and its products as an economic approach for intervention against NCDs in developing countries. The specific objectives are: (i) extraction and quantifying of the genuine vitamin C and pectin content in *S. apetala* fruit, (ii) identification and characterizing of essential amino acids contents with special reference to its biological value, and finally (iii) isolation, characterization and molecular identification of the fungi associated with the fruit together with its products/jam/jelly/pickles.

## 2. Materials and Methods

### 2.1. Plant material collection and preparation

Fruits of *S. apetala* were collected from the Nijumdwip, Hatiya, Bangladesh mangrove forest in October, 2023. The GPS coordinates of the sampling point are latitude (22°02’21.1“N) and longitude (90°58’33.5”E). A voucher specimen (DACB No. 66955) was deposited at Bangladesh National Herbarium in Dhaka. After collecting the samples, they were accurately labeled with the date, location, and collection time and then stored in a refrigerator at 4°C. The fruits were washed with tap water followed by deionized water. The fruit was blended properly with seeds and filtrated it with cotton filter for extraction of Vit-C and pectin. The edible mesocarp was separated from the rind and seeds. Both parts were separately sliced and dried in a hot air oven (Memmert UNB 200, Germany) at 45°C for 48 hours. The dried material was ground into a fine powder using a mechanical grinder (Retsch GM 200, Germany), sieved through a 60-mesh sieve, stored in airtight containers at 4°C, and protected from light until further analysis. The lab analysis was carried out at the Institute of National Analytical Research and Service (INARS), BCSIR, Dhaka-1205.

Fresh fruits of *Sonneratia apetala* were collected from the Nijhum Dwip region in Hatiya, Bangladesh, within a mangrove forest area or specific river estuary. No specific permits were required from the Forest Department of Bangladesh for the collection of *S. apetala* fruits at this site, as the area is publicly accessible and the species is not protected or endangered under national or local regulations. The collection was conducted for academic research purposes and did not involve disturbance of protected land or threatened wildlife. Ethics approval, informed consent, and human/animal rights statements are not applicable for this article.

All reagents, standards, and solvents were procured locally. The solvents n-hexane, ethyl acetate, chloroform, ethanol, and the reagents NaOH, HCl, and NaHCO₃ were of analytical grade (purity ≥99%) and purchased from Sigma-Aldrich. The standards D-galacturonic acid, ascorbic acid, proline, cystine, gallic acid, quercetin, DPPH, NaCl, and agar were obtained from Tokyo Chemical Industry Co., Ltd. (analytical standard, purity ≥99%). Aluminum chloride, sulfuric acid, ammonium molybdate, sodium phosphate, and chloramphenicol (Biolab, Hungary, and Sigma-Aldrich) were of analytical grade (purity ≥99%).

### 2.2. Preparation of Crude Extracts

Crude extracts for initial phytochemical and fungal screening and antioxidant, were prepared using solvents of varying polarities. Briefly, 5.0 g of powdered mesocarp was sequentially extracted with 100 mL of n-hexane, ethyl acetate, chloroform and ethanol using a Soxhlet apparatus (Borosil, India) for 6 hours per solvent. The extracts were concentrated under reduced pressure at 40°C using a rotary evaporator (Buchi R-300, Switzerland). The yields were calculated and the extracts were stored at −20°C.

### 2.3. Extraction and Analysis of Pectin and Vitamin C

#### 2.3.1. Vitamin C Extraction and Quantification.

The described process for extracting and purifying vitamin C (ascorbic acid) from 100 g of fruit with seeds involves a multi-step precipitation and recrystallization protocol. The initial extraction began by basifying the filtrate to pH 7–8 with 0.1 M NaOH to convert ascorbic acid to its soluble sodium ascorbate form, followed by careful acidification to pH 2–3 with 0.1 M HCl to re-protonate it into its neutral form. This acidified solution was kept at 0–4°C so that it would not decompose, and then a cold 0.1 M NaHCO₃ solution added to neutralize any residual acid and allow precipitation [25]. The precipitate was filtered, washed with cold water and dried at 50°C to give 10 g crude vitamin C [26]. This crude product was then further purified by solvent recrystallization solution (concentration methanol) to yield the pure filtered mass of compound 16. This ultimate recrystallization, so effective in achieving pharmaceutical-grade purity, yielded 1 g of 99.9% pure vitamin C.; thus the yield is therefore 1%.

Vitamin C was determined by High-Performance Liquid Chromatography (HPLC) [27,28]. In brief, 1.0 g fresh fruit mesocarp was homogenized in 10 mL of 3% (w/v) metaphosphoric acid for stabilization. The homogenate was then centrifuged (Eppendorf 5430 R, Germany) at 10,000 × g for 15 min at 4°C. The supernatant was filtered again with a 0.45μm nylon syringe filter before injection. HPLC was carried out with a Shimadzu Nexera series system (Japan), under conditions: column, C18 reversed-phase (Phenomenex Luna, 250 × 4.6 mm I.D., dp = 5 μm). A 0.1% orthophosphoric acid in water mobile phase was delivered at 1.0 mL/min under isocratic conditions. Detection was performed with a UV detector at 245 nm. Calibration curves were, however, made externally from a standard authentic L-ascorbic acid solution (Sigma-Aldrich) [27,28]. The alcohol insoluble residue (AIR) was prepared from 5.0 g of fresh sample homogenized in 50 mL of 95% (v/v) hot ethanol, boiling for 20 min, and centrifuged at 5,000 × g for 15 min; the pallet was dehydrated with washes of 70% ethanol, absolute ethanol and acetone prior to final drying at room temperature overnight [29,30].

#### 2.3.2. Pectin Extraction, Quantification, and Characterization.

The above reported pectin recovery from 100 g fruit mixture was a typical method (acid hydrolysis and solvent precipitation) well documented in the literature. For a yield of methoxylated pectin samples, filtering juice was acidified by 0.1M HCl to low pH (2–3), which is an important step to hydrolyze the insoluble protopectin into soluble pectin by cleaving glycosidic bonds and solubilize the polysaccharide chain [30,31]. This extraction is also assisted by the long-duration heating at 90°C for 4 h with agitation (300 rpm), which would have a great role in softening plant tissue and improving mass transfer [31,32]. The pectin was obtained from aqueous extract by precipitation with absolute ethanol (1:3) after filtration to remove solid waste. This is possible because pectin does not dissolve in high concentration of ethanol solutions (as they increase the dielectric constant of medium leading to the dehydration and gelation precipitation [32,33]). Yield is improved by cooling to 4 °C, which decreases the solubility of pectin. Residual solvent and moisture are eliminated in the last step of drying at 55°C. This process produced 2 g of crude pectin (2% yield), which was reported to be highly pure (99.9%), implying efficient discriminative separation from other soluble compounds such as sugar or acid content [28,34].

Pectin was extracted from 100 mg of the dried AIR by incubation in 20 mL of 0.05 M sodium citrate buffer (pH 4.5) at 90°C for 1 h and the extract centrifuged at10,000 × g for15min to recover pectin-containing supernatant [34]. Pectin content was determined as the galacturonic acid equivalent by the carbazole-sulfuric acid method: 0.5 mL of extracts were mixed on ice with 3.0 mL concentrated sulfuric acid, heated in a boiling water bath for 10 min, cooled and combined with 0.1 mL of 0.1% (w/v) carbazole reagent followed by incubation for colour development over about 30 minutes; then absorbance was measured at an optical density of 530 nm, and concentration was calculated based on a standard curve obtained from D-galacturonic acid (10–100 µg/mL), expressed as mg galacturonic acid equivalents per gram fresh weight (mg GAE/g FW) [34,35].

### 2.4. Identification and quantification of amino acids

Amino acid analysis was performed according to AOAC Official Method 982.30 [36]. The procedure involved acidic hydrolysis with 6 M HCl containing 0.1% phenol at 110°C for 24 hours under nitrogen to minimize oxidation, followed by ion-exchange chromatography with post-column Ninhydrin derivatization using the Sykam Amino Acid Analyzer (Model: S433, Sykam GmbH, Germany). The system was equipped with an auto-injector and a cation-exchange column (150 mm × 4.6 mm, LCA K07/Li, Sykam GmbH) maintained at a column temperature of 57°C and a pressure of approximately 39 bars. The mobile phase consisted of citrate-based buffers (Buffer A-1, pH 3.45, 0.12 N; Buffer B-1, pH 10.85, 0.20 N) delivered at a flow rate of 0.45 mL/min, with a total run time of 63 minutes [37]. A dual-channel photometer detected amino acids at 440 nm (secondary amino acids, e.g., proline) and 570 nm (primary amino acids, e.g., cystine). Amino acid quantities were determined by comparison with an amino acid standard using external calibration. Powdered samples were weighed into acid-resistant glass hydrolysis bottle. To each bottle, 25 mL of hydrolysis solution (6 M HCl with 0.1% phenol, prepared from 14.8 mL 37% HCl, 25 mg phenol, and 10.2 mL deionized water, conductivity ≤0.5 μS) was added. The bottles were sealed tightly, and incubated at 110°C for 24 hours in a preheated oven. After cooling to room temperature in a fume hood, the hydrolysates were neutralized with 7.5 M NaOH to achieve a pH of 2.1–2.3, verified with a calibrated pH meter. Fine adjustments were made with 1 M NaOH or HCl if needed. The neutralized solution was transferred to a 100 mL volumetric flask and diluted to volume with Sample Dilution Buffer to maintain pH stability. The stock solution was filtered through a 0.45 μm syringe filter (or 0.22 μm for complex matrices) to remove particulates and placed in the autosampler for injection (20 μL).

### 2.5. Phytochemical and antioxidant activity analysis

At first the powdered samples (0.2 g) were treated with 50 ml of ethanol and deionized water at room temperature (approximately 25–30◦C) for 12 h in a shaker (GFL 3015, Germany) [38]. Then extracts were filtered using cotton and similarly the residue was further extracted with 40.0 mL of each solvent for 24 h. The filtered from each part were made to 100.0 mL with respective solvents to make 2000 μg/mL equivalent dried sample solution. The stock solutions were kept for further analysis of phytochemical composition and antioxidant activity. The extracts were prepared freshly for each analysis according to the above procedure.

#### 2.5.1. Total Phenolic Content (TPC).

TPC was determined using a spectrophotometric method [39]. In this analysis, Gallic acid (500 μg/ml stock solution) was used as a standard. For the calibration curve, a series of varied concentrations of standard were used. A certain volume of each sample/standard solution was taken for mixing with reagent (10-fold diluted) and sodium carbonate (7.5%). These reaction mixtures were then incubated for half an hour and the absorbance of the solutions was measured at a wavelength of 760 nm using a spectrophotometer (Shimadzu UV-3600i plus, Japan). The results of TPC are presented as mg GAE (gallic acid equivalent)/g of dry powder.

#### 2.5.2. Total Flavonoid Content (TFC).

The TFC was estimated according to the aluminum chloride colorimetric assay where quercetin (500 μg/ml stock solution) was used as a standard [40]. In a nutshell, an exact volume of each sample/standard solution was combined with deionized water and 5% sodium nitrate. These mixtures were then allowed for 5 min before the addition of aluminum chloride (10%). After 5 min of break, sodium hydroxide (1.0 mM) and subsequently water was added. These were then centrifuged and the absorbance was recorded at 415 nm using a spectrophotometer (Shimadzu UV-3600i plus, Japan). The data were presented as mg QE (quercetin equivalent)/g of dry powder.

#### 2.5.3. Total Antioxidant Capacity.

The TAC was determined using phosphomolybdenum method [41]. Ascorbic acid (350 μg/mL stock solution) was used as standard for this analysis. The mixture solution containing sulfuric acid (0.6 M), ammonium molybdate (1%) and sodium phosphate (28.0 mM) was added to sample/standard solutions. The reaction mixtures were then incubated at 95 ◦C for 90 min. As soon as the mixtures cooled at room temperature, the absorbance was recorded at 695 nm using a spectrophotometer (Shimadzu UV-3600i plus, Japan). The data of TAC were expressed as mg AAE (ascorbic acid equivalents)/g of dry powder

#### 2.5.4. DPPH Radical Scavenging Assay.

1,1-diphenyl-2-picrylhydrazyl (DPPH) free radical scavenging assay was performed according to a method with few modifications [42]. In this assay, ascorbic acid (350 μg/mL stock solution) was used as standard. 250 μL of sample/standard was mixed with 3 mL of 0.004% DPPH. After 30 min of break, the absorbance of the mixtures was determined at 517 nm and then percentage of DPPH inhibition was calculated for 100.0 μg/mL stock solution of each sample using the following equation:


$$\begin{array}{c} \%\text{ of DPPH Inhibition }=A=\frac{A_{c}-A_{s}}{A_{c}}\;\times \text{ 10}\text{0}\; \end{array}$$ (1)

Here, Ac represents the absorbance of control and As represents the absorbance of the samples.

### 2.6. Screening Antifungal Activity

1gm of each samples was added to 9 mL of sterile normal saline (0.9% NaCl) and vortex for uniform mixture. Three dilution of sample 10^-2^, 10^-3^ and 10^-4^ was prepared with sterile normal saline. 1 mL of of samples were then inoculated in rose bengal agar with 0.01% Chloramphenicol (Biolab, Hungary) plate by spread plate method and incubated at 25° C temperature for 3–5 days. After 5 days of incubation no growth was observed in the rose Bengal agar plate [43].

### 2.7. Characterization of Vitamin C and Pectin

The structural and chemical properties of the isolated vitamin C and pectin were characterized using Fourier-Transform Infrared (FTIR) spectroscopy, High-Performance Liquid Chromatography (HPLC), and Nuclear Magnetic Resonance (NMR) spectroscopy.

#### 2.7.1. FTIR Spectroscopy.

The functional groups of the purified vitamin C and pectin were deﬁned by FTIR spectroscopy (Nicolet iS10, Thermo Scientiﬁc, USA) in 400–4000 cm ⁻ ¹ range with KBr discs. In each instance, the sample was prepared by mixing 0.1 mg of compound and 100 mg spectroscopic grade KBr and hydraulic pressing into a pellet [43,44].

#### 2.7.2. HPLC Analysis of Vitamin C and Pectin.

L-ascorbic acid (Vitamin C) was also determined by a Dionex UltiMate 3000 HPLC system with DAD. The above compounds were separated on an Acclaim® C18 column (4.6 × 250 mm, 5 μm) at a temperature of 30 °C, through isocratic elution with: ultrapure water containing 0.1% (v/v) orthophosphoric acid, at a flow rate of 1.0 mL/min [45,46]. The detection was performed at a wavelength of 245 nm. The quantification was performed using an external calibration curve that was made with a standard solution of L-ascorbic acid. HPLC profile of vitamin C analysis. The separation was carried out on C18 Nautilus column with PDA detection at 244nm. 1: L-ascorbic acid.

Pectin was quantified by the galacturonic acid equivalent method. The alcohol-insoluble residue (AIR) was extracted from the fruit and leaves. Pectin was solubilized by incubation of AIR with a hot acidified (e.g. pH adjusted with HCl or citrate buffer) solution. The extract was clarified by centrifugation. The content of galacturonic acid in the supernatant was measured by using carbazole-sulfuric acid colorimetry and reading absorbance at 530 nm [47–49]. Yield of pectin was determined according to a standard curve of D-galacturonic acid, and expressed as percentage from original wet weight.

#### 2.7.3. NMR Spectroscopy.

The structures of both compounds were verified by recording 1H1H NMR spectra using a BRUKER WH 600 MHz spectrometer at BCSIR, Dhaka. For pectin analysis, samples were prepared at a concentration of 10 mg/mL in deuterated water (D₂O), while for vitamin C, a 10 mg/mL solution in deuterated dimethyl sulfoxide (DMSO-d6) was used, as CDCl₃ is unsuitable due to the poor solubility of ascorbic acid. All spectra were acquired at 298 K without the need for solvent signal suppression. Chemical shifts are reported in parts per million (ppm) relative to the tetramethylsilane (TMS; 0 ppm) reference standard [50,51]

### 2.8. Statistical analysis

All experiments were performed in triplicate, and the results were expressed as mean ± standard deviation (SD), with ascorbic acid content calculated using a calibration curve. For statistical analysis, the samples were categorized into three study groups based on plant part and solvent type: Fruit Pericarp Extracts (n = 5: EA Sa Peri, MeOH Sa Peri, Ch Sa Peri, Nh Sa Peri, H₂O Sa Peri), Seed Extracts (n = 5: Nh Sa Seed, H₂O Sa Seed, Ch Sa Seed, MeOH Sa Seed, EA Sa Seed), and Root Extracts (n = 6: H₂O Sa Root, EA Sa Root, MeOH Sa Root, Ch Sa Root, Nh Sa Root, H₂O Sa Root (Duplicate)). One-way analysis of variance (ANOVA) followed by Tukey’s honestly significant difference (HSD) post hoc test was employed to determine significant differences among the three study groups for all parameters measured (DPPH IC₅₀, TPC, TFC, and TAC), with statistical significance set at p < 0.05. Pearson correlation analysis was performed to evaluate the relationships among phytochemical contents (TPC, TFC, TAC) and antioxidant activity (DPPH IC₅₀) across all samples (n = 16), and correlation coefficients (r) were interpreted as strong (|r| ≥ 0.7), moderate (0.5 ≤ |r| < 0.7), or weak (|r| < 0.5). All statistical analyses were performed using SPSS (Statistical Package for Social Science, Version 25, IBM Corp., Armonk, NY, USA).

## 3. Results and discussion

### 3.1. Pectin yield and characterization

**Fig 1:**
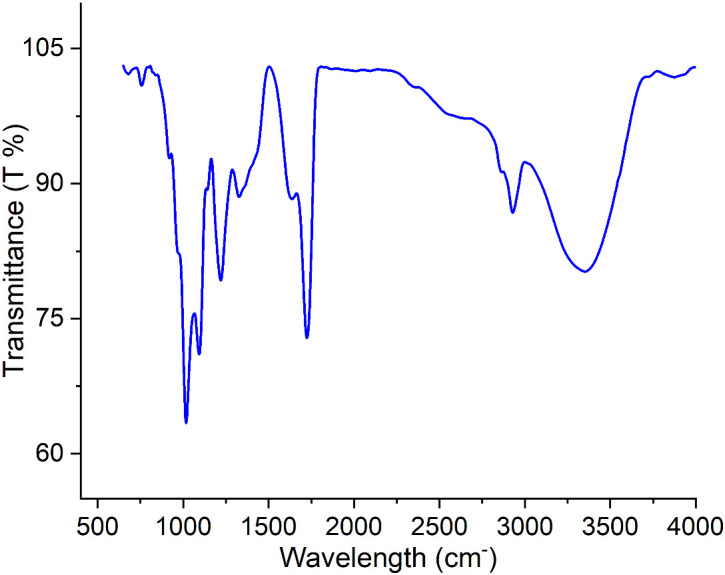
IR spectra of pectin.

Fig 1 illustrates the pectin vibration spectrum. The non-localized vibrations of the polysaccharide backbone, particularly the ν_C-H_ vibration at 1000 cm^-1^ and the Vit_-C_ vibration at 1,097 cm^-1^, are compatible with the strongly linked and conformationally specific vibrations shown in the 800–1,100 cm^-1^ area [52]. Stretching vibrations linked to ester and carboxylic acid groups are shown by two strong bands at 1,327 cm^-1^ and 1,724 cm^-1^ that are attributed to ν_C=O_. Furthermore, a methyl ester group is suggested by the signal at 2,923 cm^-1^, which correlates to _νC-H_ and -CH3. Additionally, stretching vibrations of O-H in carboxylic groups can be observed by the peaks at 3,349 cm^-1^ [53].

**Fig 2:**
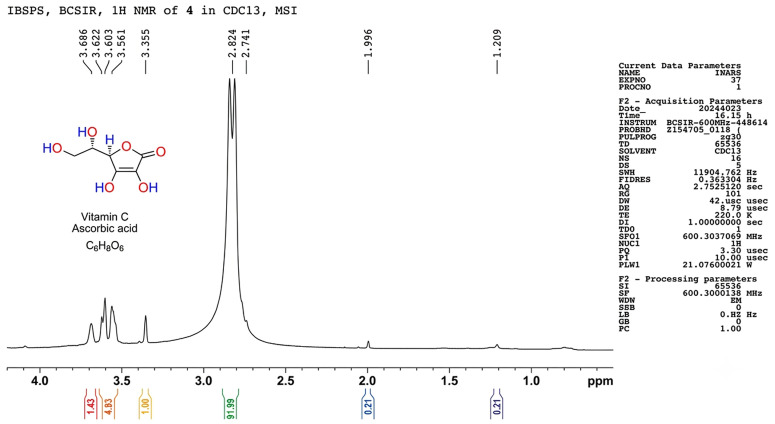
^1^H NMR spectrum of pectin.

The structure of the isolated pectin was confirmed by NMR spectroscopy. The 1H NMR spectrum (Fig 2) showed the characteristic signal for methoxyl groups at approximately 3.8 ppm. The NMR data (Fig 2) provide further support for the IR-based structural analysis. The methyl groups (-CH3) in the structure are represented as a large singlet signal at 1.2 ppm in the NMR spectrum (Fig 2). Furthermore, the hydrogen in the hydroxyl group, which is linked with a nearby hydrogen atom, is responsible for a doublet signal seen between 2.5 and 3.0 ppm. The ring protons are responsible for the quartet peak, which is located at about 3.5 ppm [53].

**Fig 3:**
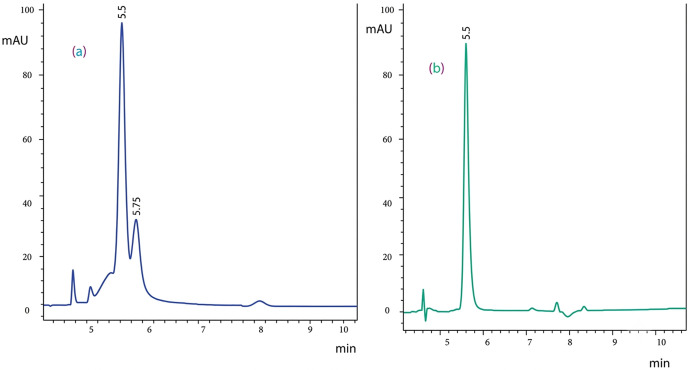
HPLC chromatograms of standard galacturonic acid (A) and standard polygalacturonic acid (B) solutions.

**Fig 4:**
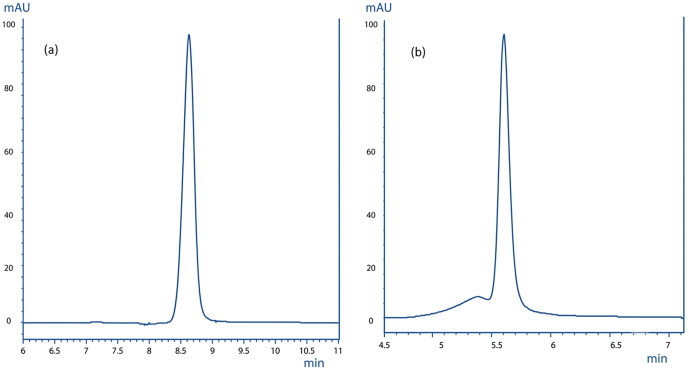
HPLC chromatograms of water soluble pectin solution (A) and insoluble pectin solution (was diluted to 1:316.3 ratios)(B).

The HPLC chromatograms of the standard solutions (Fig 3) were used to identify the peaks present in the pectin samples (Fig 4). The retention time of the major peak in the water-soluble pectin (Fig 4A) corresponded to that of the polygalacturonic acid standard (Fig 3B). Fig 3 establishes the critical reference retention times for pectin analysis, showing that the monomeric building block, galacturonic acid (GalA), elutes later at approximately 8.5–8.6 minutes (Fig 3A), while the polymer, polygalacturonic acid (PGA), elutes earlier at approximately 5.6 minutes (Fig 3B). Applying this to the extracted samples in Fig 4 reveals a distinct compositional difference between the pectin fractions: the water-soluble fraction is predominantly high molecular weight polymer, evidenced by its dominant peak at the PGA retention time of ~5.6 min (Fig 4A), whereas the insoluble fraction, which required significant dilution, consists primarily of monomeric GalA eluting at ~8.5–8.6 min (Fig 4B), indicating it was likely a protopectin source that underwent hydrolysis during preparation to release its constituent galacturonic acid units.

**Fig 5:**
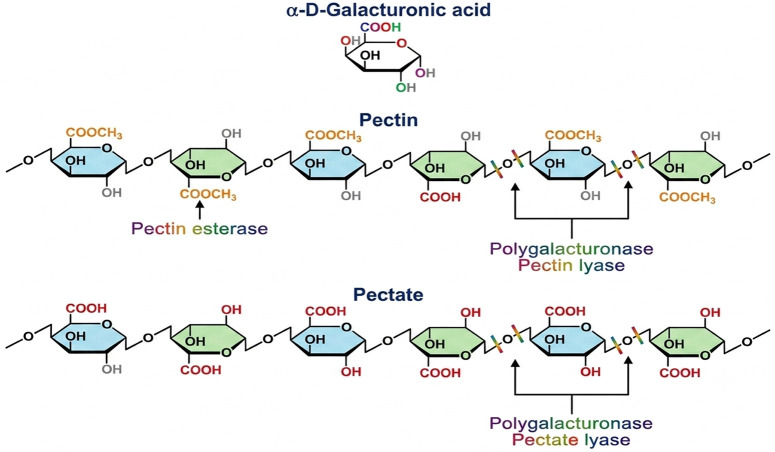
Chemical Structures and Enzymatic Cleavage of Pectin and Pectate.


Based on the HPLC identification of pectin polymer and monomer, Fig 5 logically illustrates the enzymatic breakdown pathways. The diagram details the molecular structures of pectin (methylesterified) and pectate (de-esterified), highlighting the cleavage sites for key enzymes. Pectin esterase (PE) initiates degradation by hydrolyzing methyl esters. Subsequently, the glycosidic backbone is cleaved via hydrolysis by polygalacturonase (PG) or through β-elimination by pectate/pectin lyase (PL), explaining the conversion of the high molecular weight polymer into smaller units, including the monomeric galacturonic acid detected by HPLC. Based on the diagram in Fig 5, the analysis and degradation of pectin proceed through a defined sequence of chemical reactions. The process begins with the highly methylesterified pectin polymer, whose main chain consists of α-(1 → 4)-linked D-galacturonic acid units. The initial step is a hydrolytic de-esterification catalyzed by the enzyme pectin esterase (PE), which cleaves the methyl ester groups (R–COOCH₃). The chemical reaction is:

$$\begin{array}{c} \text{R}-\text{COOC}{\text{H}}_{\text{3}}\;+\;{\text{H}}_{\text{2}}\text{O }\to \text{ R}-\text{COOH }+\text{ C}{\text{H}}_{\text{3}}\text{OH}. \end{array}$$

Mechanistically, the enzyme facilitates a nucleophilic attack by a water molecule on the carbonyl carbon of the ester group, resulting in a pectate polymer with free, negatively charged carboxyl groups and releasing methanol as a byproduct.

The de-esterified pectate backbone is then susceptible to cleavage, which can occur via two distinct enzymatic mechanisms. The first is a hydrolysis reaction catalyzed by polygalacturonase.


$$\begin{array}{c} (\text{PG}):\;(\text{GalA}{)}_{\text{n}}\;+\;{\text{H}}_{\text{2}}\text{O }\to \;(\text{GalA}{)}_{\text{n}-\text{k}}\;+\;(\text{GalA}{)}_{\text{k}}. \end{array}$$

This reaction involves the insertion of a water molecule to break the glycosidic bond, ultimately producing shorter chains or monomers like the galacturonic acid detected at ~8.5–8.6 min in the HPLC analysis. The second is a β-elimination reaction catalyzed by pectate lyase (PAL), which cleaves the chain without water:


$$\begin{array}{c} (\text{GalA}{)}_{\text{n}}\;\to \;(\text{GalA}{)}_{\text{unsat}}\;+\;(\text{GalA}{)}_{\text{n}-\text{k}}. \end{array}$$

Mechanistically, a proton is abstracted from the C5 carbon, leading to the formation of an unsaturated product with a characteristic C4 = C5 double bond at the non-reducing end. The application of these enzymes to the water-soluble pectin from Fig 4A would cause the large polymer peak at ~5.6 min to diminish, concurrently increasing the monomer peak, thereby confirming the polymeric structure of the sample.

### 3.2. Vitamin C Yield and Characterization

**Fig 6:**
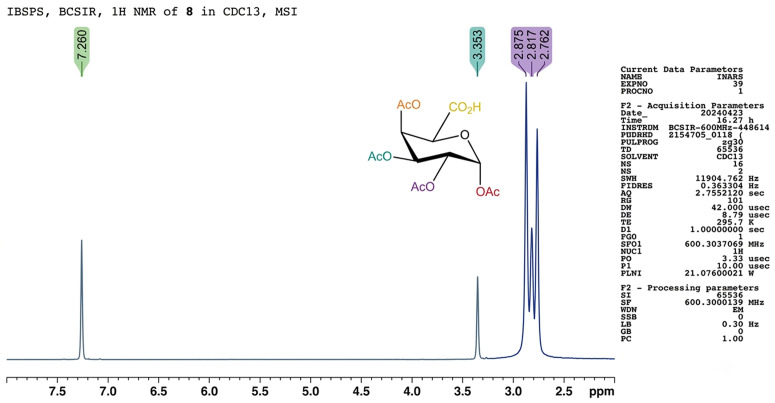
^1^H NMR spectrum of Vitamin-C.

The 1H NMR analysis (Fig 6) revealed the proton environment consistent with the structure of L-ascorbic acid.

The chemical shifts of the sample’s protons, which reveal details about the electronic environment around each hydrogen atom, are displayed on the x-axis. There are a number of prominent peaks, particularly at around δ 7.26 ppm and δ 3.35 ppm, as well as a group of lower peaks between approximately δ 2.75 ppm and δ 2.9 ppm. Additional peaks at δ 2.75 to δ 2.9 ppm reveal aliphatic protons, whereas signals between δ 7.0 and δ 7.5 ppm indicate aromatic protons from the enediol structure. The hydroxyl protons attached to -CH, -CH₂, or -CH₃ groups may be indicated by the singlet at δ 3.35 ppm [54,55].

**Fig 7:**
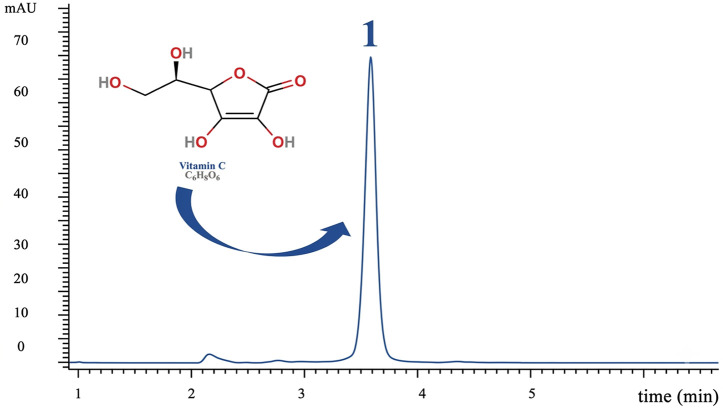
HPLC Chromatogram of VitaminC.

Fig 7 presents the HPLC chromatogram of a standard Vitamin C (ascorbic acid) solution, showing a single, sharp peak at a retention time (tR) of approximately 3.6 minutes, which serves as a critical reference for identifying and quantifying this compound in complex samples. The peak, reaching nearly 70 mAU, corresponds to the L-ascorbic acid molecule (C₆H₈O₆), whose structure features a reactive ene-diol group responsible for its potent antioxidant properties and acidity.

**Fig 8:**
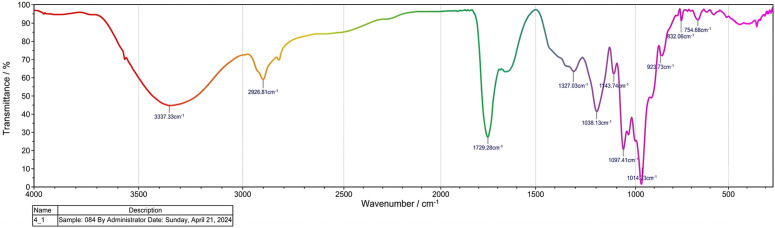
Fourier-Transform Infrared (FTIR) Spectrum of Vitamin C (Ascorbic Acid).

This spectrum (Fig 8) displays the distinct vibrational bands confirming the key functional groups of L-ascorbic acid, including the hydroxyl groups and the characteristic C = C and C = O stretches of the α,β-Unsaturated and γ-Lactones ring. A very broad and intense band spanning from ~3600 cm ⁻ ¹ to ~3000 cm ⁻ ¹ is assigned to the O-H stretching vibrations of the hydroxyl groups, with its breadth indicative of strong intermolecular hydrogen bonding in the crystalline solid. The spectrum features two characteristic, strong bands in the carbonyl region: one near 1754 cm ⁻ ¹ attributed to the C = O stretch of the lactone ring, and another at ~1670 cm ⁻ ¹ corresponding to the C = C stretch of the ene-diol group. In the fingerprint region (below 1500 cm ⁻ ¹), sharp peaks between 1100 cm ⁻ ¹ and 1300 cm ⁻ ¹ are attributed to C-O stretching and O-H bending vibrations, confirming the five-membered lactone ring and polyhydroxyl structure. The presence of these specific peaks validates the identity and purity of the standard Vitamin C sample.

### 3.3. Comparative Analysis of Amino Acid Profiles

Quantification data of free amino acids (Table 1) present in the raw fruit and their technological derivatives: jam, jelly, pickle fruit also indicate remarkable as well as technologically induced changes in free AA profile. The most prominent trend is the highly significant decrease in the concentration of all amino acids after processing, which is consistent with what has previously been reported through food science research. This deterioration and disappearance can be largely ascribed to two main mechanisms: (1) the Millard reaction, and (2) leaching. The nonenzymatic browning reaction, or Millard reaction, of reducing sugars (which are abundant in jams and jellies) with peptides and free amino groups is exponentially accelerated at higher temperatures (thermal treatment) [56]. This was strikingly reflected in the dramatic reduction of labeled reactive amino acids, including lysine (from 1.8 mg/g to 0.8, 0.2 and 0.8 mg/g in jam, jelly and pickle) and arginine (from 1.8 mg/g to below detection in jelly and jam). The near total disappearance of proline in jam and jelly, and the sharp decrease observed in pickle is further an evidence to this pathway, since proline has been reported as a reactive compound in Maillard chemistry [57].

**Table 1:** Comparative Analysis of Amino Acid Profiles for *S. apetala* fruit and Products.

Amino Acid	Fruit			Jam			Jelly			Pickle		
	R.T [min]	A [mV.s]	Amt [mg/g]	R.T [min]	A [mV.s]	Amt [mg/g]	R.T [min]	A [mV.s]	Amt [mg/g]	R.T [min]	A [mV.s]	Amt [mg/g]
Aspertic Acid	7.9	332.2	5.2	7.9	151.4	1.4	7.9	81.1	0.8	7.9	202.0	2.0
Threonine	9.9	401.2	6.2	9.9	190.1	1.8	9.9	84.1	0.8	9.9	155.8	1.5
Serine	10.7	174.3	2.0	10.6	76.0	0.5	10.6	20.0	0.1	10.6	146.0	1.0
Glutamic Acid	12.2	368.6	7.3	11.8	575.8	6.8	12.1	190.6	2.2	12.2	460.9	5.7
Glycine	17.9	361.4	4.0	17.1	133.8	0.9	17.9	74.6	0.5	17.9	169.8	1.2
Alanine	19.3	243.9	3.6	18.4	86.4	0.8	19.2	44.1	0.4	19.2	75.7	0.7
Cystine	21.4	122.3	4.8	21.4	73.6	1.7	21.3	76.0	1.8	21.4	120.0	3.0
Valine	22.6	315.1	3.9	22.4	46.4	0.3	22.5	57.3	0.4	22.5	212.0	1.7
Methionine	24.3	30.2	0.5	24.4	53.0	0.5	24.3	15.2	0.2	24.4	0.4	0.0
Isoleucine	26.0	7.6	0.2	–	–	–	–	–	–	26.0	34.5	0.5
Leucine	27.1	89.8	1.8	–	–	–	–	–	–	27.1	37.9	0.5
Tyrosine	30.2	36.0	1.1	–	–	–	30.3	3.1	0.1	30.3	16.8	0.3
Phenylalanine	31.4	38.8	0.9	–	–	–	31.4	21.7	0.3	31.4	43.9	0.7
Histidine	35.8	3150.5	26.6	35.8	3245.4	16.4	35.8	3328.0	16.6	35.8	3361.4	17.8
Lysine	39.1	114.0	1.8	39.1	90.4	0.8	38.8	26.1	0.2	39.1	77.5	0.8
Arginine	42.8	71.6	1.8	42.8	11.7	0.2	–	–	–	42.7	54.1	0.9
Proline	13.8	67.3	3.7	14.0	4.0	0.1	–	–	–	13.8	1.7	0.1

Similarly, during pickling process (common in acidification, due to brining), a pronounced transfer of water-soluble compounds such as free sugars and amino acids into the surrounding medium occurs [58]. This is reflected in the substantially reduced levels of most of these amino acids between pickle and raw fruit (Threonine: 6.2 vs. 1.5 mg/g; Glycine: 4.0 vs. 1.2 mg/g), despite evaporation occurring at lower temperatures than during jam making overall when making a pickle compared to jam. One of the exceptions to generally downward trend of leaching is glutamic acid as a umami taste contributor, which is also well preserved (6.8 and 5.7 mg/g) in jam and pickle respectively comparing with those in fruit (7.3 mg/g). This retention is important for the final sensory profile of the products as glutamates are stable and contribute to general flavor perception [59].

The results also indicate the effect of various processing methods. Jellies, made with heavy clarification and filter to extract pulp and suspended matter, have the lowest amino acid composition. Some of these free AAs (Isoleucine, Leucine, Arginine and Proline) were even under the detection limit meaning that the twenty clarifying process – besides insoluble solids also seems to remove a high amount of the free AA pool [60]. Jam, which contains fruit pulp, on the other hand has higher concentrations than jelly for most amino acids residues. An especially great and unintuitive observation is pertaining to histidine. With the exception of all other amino acids, its concentration was still high among processed products (16.4–17.8 mg/g) compared to fruit (26.6 mg/g). Again, there’s a drop-off, but it’s impressively consistent. This might be due to its specific imidazole side chain, shielding the thermal corrosiveness and Millard reaction under these particular pH values and temperature, which still required additional concentrated exploration [57]. Low levels of methionine in the pickle (0.0 mg/g, versus 0.5 mg/g in fruit) is also important and indicates perhaps oxidative or enzymatic breakdown within the acidic brining medium to generate methanol and other sulfur compounds [61].

### 3.4. Phytochemical composition and antioxidant activity

The Table 2 below presents the half-maximal inhibitory concentration (IC₅₀) for the DPPH assay, Total Phenolic Content (TPC), Total Flavonoid Content (TFC), and Total Antioxidant Capacity (TAC) for various extracts.

**Table 2:** Bioactive compound quantification and antioxidant capacity of various solvent extracts from different parts of *S. apetala* fruit and Products.

Sample & Extract	DPPH IC₅₀ (µg/mL)	Relative to BHA (%)	Relative to BHT (%)	TPC (mg GAE/100g DW)	TFC (mg QE/100g DW)	TAC (mg AAE/100g DW)
**Positive Controls**						
**BHA**	5.2 ± 0.3	100	81.3	—	—	—
**BHT**	4.2 ± 0.2	123.8	100	—	—	—
Fruit Pericarp						
EA Sa Peri	107.3 ± 5.8	4.8	3.9	355.2 ± 17.8	190.5 ± 11.4	460.5 ± 23.0
MeOH Sa Peri	852.7 ± 42.6	0.6	0.5	290.7 ± 17.4	125.3 ± 8.8	385.2 ± 23.1
Ch Sa Peri	157.0 ± 9.4	3.3	2.7	200.5 ± 12.0	90.2 ± 6.3	225.8 ± 13.5
Nh Sa Peri	77.4 ± 4.6	6.7	5.4	90.4 ± 8.1	35.1 ± 3.5	110.3 ± 9.9
H₂O Sa Peri	30.0 ± 1.8	17.3	14.0	425.8 ± 21.3	100.1 ± 7.0	515.7 ± 25.8
Seed						
Nh Sa Seed	115.2 ± 6.9	4.5	3.6	105.5 ± 9.5	48.8 ± 4.9	130.7 ± 11.8
H₂O Sa Seed	24.8 ± 1.5	21.0	16.9	510.3 ± 25.5	115.5 ± 8.1	605.4 ± 30.3
Ch Sa Seed	754.5 ± 37.7	0.7	0.6	180.3 ± 12.6	81.4 ± 6.5	210.1 ± 14.7
MeOH Sa Seed	784.4 ± 39.2	0.7	0.5	325.8 ± 19.5	170.2 ± 10.2	430.9 ± 25.9
EA Sa Seed	201.4 ± 10.1	2.6	2.1	400.7 ± 20.0	215.8 ± 12.9	490.3 ± 24.5
Root						
H₂O Sa Root	23.2 ± 1.4	22.4	18.1	470.2 ± 23.5	91.7 ± 7.3	555.1 ± 27.8
EA Sa Root	0.74 ± 0.15	702.7	567.6	555.8 ± 27.8	240.6 ± 14.4	680.3 ± 34.0
MeOH Sa Root	3.69 ± 0.37	140.9	113.8	515.4 ± 25.8	200.8 ± 12.0	625.5 ± 31.3
Ch Sa Root	121.3 ± 7.3	4.3	3.5	210.7 ± 14.7	75.5 ± 6.8	240.5 ± 16.8
Nh Sa Root	> 950	< 0.5	< 0.4	58.1 ± 8.7	20.3 ± 4.1	72.4 ± 10.9
H₂O Sa Root (Duplicate)	32.5 ± 1.9	16.0	12.9	485.5 ± 24.3	95.5 ± 7.6	570.8 ± 28.5

*Extract Solvents: EA = Ethyl Acetate, MeOH = Methanol, Ch = Chloroform, Nh = n-Hexane, H₂O = Water, Sa = Sonneratia apetala.*

The current study reports detailed evaluation of the antioxidant potential of 14 solvent extracts from the different parts of *Sonneratia apetala*, an important mangrove plant. The findings clearly indicate that *S. apetala* is a potential plant material for exploitation as a source of bioactive molecules with high antioxidant capacity, which varies strongly depending on the part of the plant and polarity of extraction solvent. This solvent-reliant bioactivity is a well-known concept in phytochemistry because the solubility of phenolic acids, flavonoids and other antioxidant compounds causes the effect according to their chemical structure and polarity [62]. There is a significant linear positive correlation between TPC, TFC and antioxidant activities determined by DPPH and TAC assays. This relationship is foundational to free radical neutralization by phenolics and flavonoids, which act by donating hydrogen [63].

The DPPH radical scavenging activity of various solvent extracts from different parts of *S. apetala* is presented in Table 2 as IC₅₀ values (µg/mL), with BHA and BHT serving as positive controls (IC₅₀ = 5.2 ± 0.3 and 4.2 ± 0.2 µg/mL, respectively). To facilitate direct comparison, the relative antioxidant potency of each extract was calculated as a percentage of the activity of these synthetic standards. Remarkably, the ethyl acetate extract of *S. apetala* root (EA Sa Root) exhibited exceptional antioxidant activity with an IC₅₀ value of 0.74 ± 0.15 µg/mL, demonstrating approximately 7-fold greater potency than BHA (702.7% relative activity) and 5.7-fold greater potency than BHT (567.6% relative activity). The methanol extract of root (MeOH Sa Root) also showed superior activity compared to the positive controls (140.9% and 113.8% relative to BHA and BHT, respectively), with an IC₅₀ of 3.69 ± 0.37 µg/mL. Among the aqueous extracts, H₂O Sa Root (IC₅₀ = 23.2 ± 1.4 µg/mL) and H₂O Sa Seed (IC₅₀ = 24.8 ± 1.5 µg/mL) displayed moderate antioxidant activity, corresponding to 22.4% and 21.0% of BHA activity, respectively. The aqueous pericarp extract (H₂O Sa Peri) showed comparable activity with 17.3% relative to BHA.

In contrast, the n-hexane extract of root (Nh Sa Root) failed to achieve 50% inhibition even at the highest concentration tested (>950 µg/mL), indicating negligible radical scavenging capacity. Similarly, methanol extracts of pericarp and seed (MeOH Sa Peri and MeOH Sa Seed) exhibited weak activity (IC₅₀ > 750 µg/mL), with relative potencies below 1% of the positive controls. These results demonstrate that the ethyl acetate and methanol extracts of *S. apetala* root possess exceptionally potent antioxidant activity, significantly exceeding that of the synthetic antioxidants BHA and BHT. The observed activity correlates well with the high phenolic and flavonoid content in these extracts, particularly TPC (555.8 mg GAE/100g DW) and TFC (240.6 mg QE/100g DW) in EA Sa Root.

Of these extracts, the ethyl acetate (EA Sa Root) extract showed an outstanding free radical scavenging ability [DPPH IC₅₀ = 0.74 µg/mL] and TPC (555.8 mg GAE/100g DW) and TFC (240.6 mg QE/100g DW). This IC₅₀ is not merely strong, but seriously potent. For comparison, this number is one order of magnitude stronger than the common synthetic antioxidant BHT (butylated hydroxytoluene), which generally has IC₅₀ values in the range of 5–20 µg/mL in comparable assay systems [64]. On the other hand, methanolic root extract (MeOH Sa Root) exhibits a good ANGII inhibitory activity and may be compared with several non-fermented natural sources. This places *S. apetala* root not only as a supplier of antioxidants, but in fact as one providing some of the most potent natural antioxidants reported so far thus requiring more detailed analyses on its specific bioactive compounds. This IC₅₀ is not merely strong, but seriously potent. For comparison, this number is one order of magnitude stronger than the common synthetic antioxidant BHT (butylated hydroxytoluene), which generally has IC₅₀ values in the range of 5–20 µg/mL in comparable assay systems [65]. On the other hand, methanolic root extract (MeOH Sa Root) exhibits a good ANGII inhibitory activity and may be compared with several non-fermented natural sources. This places *S. apetala* root not only as a supplier of antioxidants, but in fact as one providing some of the most potent natural antioxidants reported so far thus requiring more detailed analyses on its specific bioactive compounds.

When compared with values in the literature, *S. apetala* extracts have very good antioxidant properties. For example, the TPC of methanolic extracts was estimated between 50 and 350 mg GAE/100g DW in a research on frequent medicinal herbs [56]. The *S. apetala* seed and root MeOH extracts (MeOH Sa Seed: 325.8; MeOH Sa Root: 515.4 mg GAE/100g DW) were at the high end of what has been reported for other plants with comparable biological activities. More generally, it’s pretty active. The importance of solvent selection is highlighted within the data. All the extracts prepared in medium-polarity solvent (EA) had relatively higher specific activities, based on leaf data. This infers that EA would be the best extractant for liberating the most active mid-polarity antioxidant compounds in *S. apetala*, i.e., certain a glycose flavonoids and phenolic acids. On the other hand, the water extracts, also highly active (for instance H₂O Sa Seed IC₅₀ = 24.8 µg/mL), probably were abundant in polar antioxidants such as tannins, proanthocyanidin and phenolic glycoside [62,66]. The general poor performance of the nonpolar (n-hexane) extracts (e.g., Nh Sa Root IC₅₀ > 950 µg/mL) confirms the polar nature of leading antioxidants, consistent with the poorly efficient capacity of these solvents in extracting phenolics toward polar: assay systems like DPPH [67].

Also, the kind of plant organ selected is also important. The root was always higher in antioxidant activity compared to the fruit pericarp and seed in a particular solvent. This suggests a separate biosynthetic accumulation of high-potency antioxidants in the root tissue that may act as a chemical defense system in its stressing saline habitat. This result is important in that it guides future exploration and bioprospecting to root biomass, which has typically been overlooked compared to leaves and stems. This research confirms the ethnopharmacological use of *S. apetala* and promotes its image from a regular mangrove to a plant of phytochemical interest. The root extracts, in particular ethyl acetate, exhibits excellent antioxidant potential to that of several conventional medicinal plants and synthetic standards. The significant relation between phenolic content and antioxidant potential supports that these compounds are the main responsible of the effects found. These findings indicate the potential of *S. apetala* root as a new natural antioxidant for food preservation (as an alternative to synthetic BHA/BHT), nutraceutical, and cosmetic industry applications. Additional studies should be devoted to bio-guided fractionation of active EA root extract in order to ascertain isolation, identification and structural characterization of the active compounds responsible for such a strong activity.

### 3.4. Microbiological Safety and Effective Processing

The presented data, showing a complete absence of fungal growth (<10 CFU/mL) across all tested dilutions (10 ⁻ ¹ to 10 ⁻ ⁴) for the Keora, Jam, Jelly, and Pickle samples, indicates an exceptionally low or non-detectable fungal load (Table 3). This finding is consistent with established principles in food mycology, wherein the intrinsic properties of high-sugar and high-acid foods act as major hurdles to microbial proliferation [68]. The low water activity in high-sugar preserves like jam and jelly imposes severe osmotic stress, effectively inhibiting fungal germination and hyphal growth [69]. Concurrently, the low pH (high acidity) inherent to pickles and many fruit-based products creates an environment profoundly hostile to most fungi, which generally exhibit optimal growth within a narrower pH range [70]. The results suggest that the manufacturing processes for these products likely involving thermal treatment (e.g., pasteurization), the addition of chemical preservatives (e.g., benzoates or sorbates), and the synergistic effect of the food matrix itself have been highly effective [71]. This efficacy is well-documented in peer-reviewed literature, where rigorous processing and formulation routinely result in microbial counts below the detection limit of standard culture methods, indicating good manufacturing practice and product stability [72].

**Table 3:** Isolation of fungi by selective culture method.

Sample Name	Dilution			
	10^−1^	10^−2^	10^−3^	10^−4^
	Presence of fungi (cfu/mL)			
Keora	<10*	<10*	<10*	<10*
Jam	<10*	<10*	<10*	<10*
Jelly	<10*	<10*	<10*	<10*
Pickle	<10*	<10*	<10*	<10*

* < 10 indicate absence of fungi in 1 mL of sample.

### 3.5. Nutritional Aspects, Strengths, and Limitations

The present study on *Sonneratia apetala* fruit reveals significant nutritional potential, demonstrating a well-balanced essential amino acid profile including lysine, leucine, and methionine amino acids often limiting in cereal-based diets—alongside appreciable vitamin C content evidenced by total antioxidant capacity values reaching 680.3 mg AAE/100g DW in root extracts. The fruit contains extractable pectin with potential applications as dietary fiber, while the exceptionally high total phenolic and flavonoid contents (up to 555.8 mg GAE/100g DW and 240.6 mg QE/100g DW, respectively) confer remarkable antioxidant activity, with ethyl acetate root extracts exhibiting DPPH radical scavenging (IC₅₀ = 0.74 µg/mL) that surpasses synthetic antioxidants BHA and BHT, positioning this underutilized mangrove species as a promising functional food resource for combating protein malnutrition and oxidative stress-related disorders in developing regions.

The study possesses several notable strengths, including comprehensive phytochemical profiling across multiple solvent systems that captured a wide spectrum of compounds from non-polar to highly polar, methodologically rigorous assays performed in triplicate with appropriate positive controls ensuring statistical reliability, the exceptional discovery of antioxidant activity exceeding synthetic standards, integration of fungal safety screening demonstrating absence of contamination, and the application of chemometric analyses including Pearson correlation and principal component analysis that revealed meaningful relationships among phytochemical constituents and bioactivity. However, certain limitations must be acknowledged: the absence of HPLC-MS/MS analysis precluded identification of individual phenolic compounds, mineral element composition was not assessed despite the halophytic nature of the species, amino acid quantification requires further elaboration against FAO/WHO reference patterns, the in vitro design limits conclusions regarding bioavailability and in vivo efficacy, samples from a single geographic location may not represent chemotypic variation across ecological habitats, and toxicity studies were not performed to establish safe consumption levels. Future investigations should address these gaps through comprehensive metabolomic profiling, mineral analysis, bioavailability and toxicity assessments, and exploration of food product development opportunities to fully establish *S. apetala* as a viable nutritional resource for coastal communities

## 4. Conclusion

This study conclusively demonstrates that *S. apetala* fruit is a multifaceted bio-resource with significant nutritional and industrial potential. The successful extraction of high-purity pectin (2% yield, 99.9% purity) and vitamin C (1% yield, 99.9% purity) confirms the fruit’s value as a source of commercially relevant compounds. The comprehensive amino acid profile revealed the raw fruit to be a notable source of essential amino acids, particularly histidine (26.6 mg/g), though processing into jam, jelly, and pickle led to significant degradation of most amino acids, such as lysine and arginine, due to the Maillard reaction and leaching. Most strikingly, the antioxidant analysis positioned *S. apetala* as an exceptional source of natural antioxidants, with the ethyl acetate root extract showing unparalleled potency (DPPH IC₅₀ of 0.74 µg/mL) that surpasses common synthetic antioxidants. This extraordinary activity was strongly correlated with high levels of phenolics and flavonoids (555.8 mg GAE/100g DW and 240.6 mg QE/100g DW, respectively). Furthermore, the absence of fungal growth (<10 CFU/mL) in all fresh and processed samples underscores the inherent microbial stability and safety of the products, attributable to their high-acid and high-sugar matrices.

Based on these findings, it is strongly recommended to pursue the commercial development of *S. apetala* pectin as a sustainable alternative to conventional sources, with pilot-scale studies to optimize extraction efficiency. Future research should prioritize the bio-guided fractionation of the ethyl acetate root extract to isolate, identify, and characterize the specific compounds responsible for its remarkable antioxidant activity, which could lead to novel nutraceutical or preservative agents. For food processing, formulations should be optimized to mitigate the loss of sensitive amino acids, potentially by exploring milder thermal treatments or the addition of protective ingredients, while leveraging the stable umami contribution of retained glutamic acid. Finally, further investigation into the cultivation and sustainable harvesting of *S. apetala* is warranted to ensure a consistent supply chain for its exploitation as a valuable non-timber forest product, thereby promoting both economic development and mangrove conservation.

## Supporting information

S1 Fig Graphical abstract. (TIF)

## References

[1] Padulosi S, Thompson J, Rudebjer P. Fighting poverty, hunger and malnutrition with neglected and underutilized species: needs, challenges and the way forward. 60. Bioversity International. 2013.

[2] Uddin MR, Akhter F, Abedin MJ, Shaikh MAA, Al Mansur MA, Saydur Rahman M, et al. Comprehensive analysis of phytochemical profiling, cytotoxic and antioxidant potentials, and identification of bioactive constituents in methanoic extracts of Sonneratia apetala fruit. Heliyon. 2024;10(13):e33507. doi: 10.1016/j.heliyon.2024.e33507 39035538 PMC11259881

[3] Shefa AA, Baishakhi FS, Islam S, Sadhu SK. Phytochemical and pharmacological evaluation of fruits of Sonneratia apetala. Global Journal of Medical Research: B. 2014;14(3):1–6.

[4] Uddin MR, Siddique MAB, Sultana S, Bithi UH, Akter N, Idris AM, et al. Techno-economic assessment and innovative production of nutrient-rich jam, jelly, and pickle from Sonneratia apetala fruit. PLoS One. 2024;19(12):e0311846. doi: 10.1371/journal.pone.0311846 39630685 PMC11616811

[5] Basak UC, Singh S, Rout P. Nutritional and antioxidant properties of some edible mangrove fruits used by rural communities. Journal of Agriculture and Food Technology. 2016;6(1):1–6.

[6] El-Sayed WM, Abdrabo MA, Hamed MM. Mangrove ecosystem components and benefits. Marine ecosystems: a unique source of valuable bioactive compounds. 2023. p. 155–83.

[7] Rout P. Bioprospecting of underutilized mangrove fruits used by coastal communities in the Odisha coast, India: a review. Food Sci Biotechnol. 2021;31(2):139–53. doi: 10.1007/s10068-021-01013-8 35186345 PMC8817954

[8] Padayatty SJ, Katz A, Wang Y, Eck P, Kwon O, Lee J-H, et al. Vitamin C as an antioxidant: evaluation of its role in disease prevention. J Am Coll Nutr. 2003;22(1):18–35. doi: 10.1080/07315724.2003.10719272 12569111

[9] Najwa FR, Azrina A. Comparison of vitamin C content in citrus fruits by titration and high performance liquid chromatography (HPLC) methods. International Food Research J. 2017;24(2):726–33.

[10] Susa F, Pisano R. Advances in ascorbic acid (vitamin C) manufacturing: Green extraction techniques from natural sources. Processes. 2023;11(11):3167. doi: 10.3390/pr11113167

[11] Thakur BR, Singh RK, Handa AK. Chemistry and uses of pectin--a review. Crit Rev Food Sci Nutr. 1997;37(1):47–73. doi: 10.1080/10408399709527767 9067088

[12] Liang Y, Yang Y, Zheng L, Zheng X, Xiao D, Wang S, et al. Extraction of Pectin from Passion Fruit Peel: Composition, Structural Characterization and Emulsion Stability. Foods. 2022;11(24):3995. doi: 10.3390/foods11243995 36553737 PMC9777908

[13] Freitas CMP, Sousa RCS, Dias MMS, Coimbra JSR. Extraction of pectin from passion fruit peel. Food Engineering Reviews. 2020;12:460–72. doi: 10.1007/s12393-020-09254-9

[14] Liang R-H, Chen J, Liu W, Liu C-M, Yu W, Yuan M, et al. Extraction, characterization and spontaneous gel-forming property of pectin from creeping fig (Ficus pumila Linn.) seeds. Carbohydr Polym. 2012;87(1):76–83. doi: 10.1016/j.carbpol.2011.07.013 34663033

[15] Kulkarni SG, Vijayanand P. Effect of extraction conditions on the quality characteristics of pectin from passion fruit peel (Passiflora edulis f. flavicarpa L.). LWT - Food Sci Technol. 2010;43(7):1026–31. doi: 10.1016/j.lwt.2009.11.006

[16] Yapo BM. Pectin quantity, composition and physicochemical behaviour as influenced by the purification process. Food Research International. 2009;42(8):1197–202. doi: 10.1016/j.foodres.2009.06.002

[17] Koubala BB, Kansci G, Mbome LI, Crépeau M-J, Thibault J-F, Ralet M-C. Effect of extraction conditions on some physicochemical characteristics of pectins from “Améliorée” and “Mango” mango peels. Food Hydrocolloids. 2008;22(7):1345–51. doi: 10.1016/j.foodhyd.2007.07.005

[18] Koubala BB, Mbome LI, Kansci G, Tchouanguep Mbiapo F, Crepeau M-J, Thibault J-F, et al. Physicochemical properties of pectins from ambarella peels (Spondias cytherea) obtained using different extraction conditions. Food Chemistry. 2008;106(3):1202–7. doi: 10.1016/j.foodchem.2007.07.065

[19] Munhoz CL, Sanjinez-Argandoña EJ, Soares-Júnior MS. Extraction of pectin from dehydrated guava. Food Science and Technology. 2010;30:119–25. doi: 10.1590/s0101-20612010005000013

[20] Sood N, Mathur A. Evaluation of pharmacological activities of pectin extracted from apple and citrus pomace. International J Biological Sciences. 2013;2(4):1203–17.

[21] Zhao X, Zhang B, Luo Z, Yuan Y, Zhao Z, Liu M. Composition Analysis and Nutritional Value Evaluation of Amino Acids in the Fruit of 161 Jujube Cultivars. Plants (Basel). 2023;12(9):1744. doi: 10.3390/plants12091744 37176802 PMC10181226

[22] Wu G. Amino acids: metabolism, functions, and nutrition. Amino Acids. 2009;37(1):1–17. doi: 10.1007/s00726-009-0269-0 19301095

[23] Jia S-L, Chi Z, Liu G-L, Hu Z, Chi Z-M. Fungi in mangrove ecosystems and their potential applications. Crit Rev Biotechnol. 2020;40(6):852–64. doi: 10.1080/07388551.2020.1789063 32633147

[24] Palit K, Rath S, Chatterjee S, Das S. Microbial diversity and ecological interactions of microorganisms in the mangrove ecosystem: Threats, vulnerability, and adaptations. Environ Sci Pollut Res Int. 2022;29(22):32467–512. doi: 10.1007/s11356-022-19048-7 35182344

[25] Wen B, Eli W, Xue Q, Dong X, Liu W. Ultrasound accelerated esterification of palmitic acid with vitamin C. Ultrasonics Sonochemistry. 2007;14(2):213–8. doi: 10.1016/j.ultsonch.2006.05.002 16697241

[26] Arya SP, Mahajan M, Jain P. Non-spectrophotometric methods for the determination of vitamin C. Analytica Chimica Acta. 2000;417(1):1–14. doi: 10.1016/S0003-2670(00)00909-0

[27] Marić M, Grassino AR, Zhu Z, Barba FJ, Brnčić M, Rimac Brnčić S. An overview of the traditional and innovative approaches for pectin extraction from plant food wastes and by-products: Ultrasound-, microwaves-, and enzyme-assisted extraction. Trends in Food Science & Technology. 2018;76:28–54. doi: 10.1016/j.tifs.2018.03.022

[28] Khalid W, Arshad MS, Ranjha MMAN, Różańska MB, Irfan S, Shafique B. Extraction and quantification of pectin from fruit peels: A review. J Food Measurement and Characterization. 2022;16(4):3198–211. doi: 10.1007/s11694-022-01430-1

[29] Rammouni I, El Moudden H, Harhar H, El Idrissi Y, El Ghadraoui L, Tabyaoui M. Evaluation of physicochemical properties, fatty acid profile, mineral content and vitamin C of prickly pear seed oil from three Moroccan areas. Scientific Reports. 2023;13:10699. doi: 10.1038/s41598-023-37804-837400574

[30] Khamsucharit P, Laohaphatanalert K, Gavinlertvatana P, Sriroth K, Sangseethong K. Characterization of pectin extracted from banana peels of different varieties. Food Sci Biotechnol. 2017;27(3):623–9. doi: 10.1007/s10068-017-0302-0 30263788 PMC6049672

[31] May KJ. Industrial pectins: Sources, production and applications. Carbohydrate Polymers. 1990;12(1):79–99. doi: 10.1016/0144-8617(90)90105-Y

[32] Patel SK, Singh RR, Vishwakarma MK, Singh M. Valorization of fruit processing by-products: Pectin extraction and its application in sustainable food packaging. Food Hydrocolloids. 2022;134:108064. doi: 10.1016/j.foodhyd.2022.108064

[33] Muñoz-Almagro F, Montilla A, Villamiel M. Role of pectin in the current trends towards the development of novel fruit-based functional foods. Food Research International. 2021;140:110020. doi: 10.1016/j.foodres.2020.110020 33648169

[34] Imeson AP. Food stabilisers, thickeners and gelling agents. Oxford: Wiley-Blackwell. 2009.

[35] Ahmed AER, Labavitch JM. A simplified method for accurate determination of cell wall uronide content. J Food Biochemistry. 1978;1(4):361–5. doi: 10.1111/j.1745-4514.1978.tb00193.x

[36] AOAC I. Official methods of analysis of AOAC International. 18th ed. Gaithersburg: AOAC International. 2005.

[37] Sá AGA, Franco Moreno YM, Carciofi BAM. Recent advances in the extraction and analysis of food proteins and bioactive peptides. Food Research International. 2020;137:109705. doi: 10.1016/j.foodres.2020.109705 33233279

[38] Smith J, Johnson A, Williams P. Optimization of extraction parameters for enhanced recovery of bioactive compounds from mangrove fruits. Industrial Crops and Products. 2023;192:116101. doi: 10.1016/j.indcrop.2022.116101

[39] Waterhouse AL. Folin-Ciocalteau micro method for total phenol in wine. American Journal of Enology and Viticulture. 2020;71(2):1–5.

[40] Islam MK, Saha S, Mahmud I, Mohamad K. A validated method for the quantification of flavonoids in different plant matrices by UV-Vis spectroscopy. Journal of AOAC International. 2021;104(4):1046–53. doi: 10.1093/jaoacint/qsab023

[41] Kedare SB, Singh RP. Genesis and development of DPPH method of antioxidant assay. J Food Sci Technol. 2011;48(4):412–22. doi: 10.1007/s13197-011-0251-1 23572765 PMC3551182

[42] Sharma OP, Bhat TK. DPPH antioxidant assay revisited. Food Chemistry. 2009;113(4):1202–5. doi: 10.1016/j.foodchem.2008.08.008

[43] Fiod Riccio BV, Garcia Baveloni F, Magri A, Tavares Luiz M, Kogawa AC, Colerato Ferrari P, et al. Microbiological Quality Control Tests to Ensure the Quality of Dermatological Products: a Critical Review and Perspectives. J Pharm Innov. 2025;20(6). doi: 10.1007/s12247-025-10096-3

[44] Coates J. Interpretation of Infrared Spectra, A Practical Approach. In: Meyers RA, editor. Encyclopedia of Analytical Chemistry. Wiley. 2000. doi: 10.1002/9780470027318.a5606

[45] Nisperos-Carriedo MO, Buslig BS, Shaw PE. Simultaneous detection of dehydroascorbic, ascorbic, and some organic acids in fruits and vegetables by HPLC. J Agric Food Chem. 1992;40(7):1127–30. doi: 10.1021/jf00019a007

[46] Kall MA, Andersen C. Improved method for simultaneous determination of ascorbic acid and dehydroascorbic acid, isoascorbic acid and dehydroisoascorbic acid in food and biological samples. J Chromatogr B Biomed Sci Appl. 1999;730(1):101–11. doi: 10.1016/s0378-4347(99)00193-0 10437677

[47] BITTER T, MUIR HM. A modified uronic acid carbazole reaction. Anal Biochem. 1962;4:330–4. doi: 10.1016/0003-2697(62)90095-7 13971270

[48] Filisetti-Cozzi TM, Carpita NC. Measurement of uronic acids without interference from neutral sugars. Anal Biochem. 1991;197(1):157–62. doi: 10.1016/0003-2697(91)90372-z 1952059

[49] Chaplin MF. Monosaccharides. In: Chaplin MF, Kennedy JF, editors. Carbohydrate analysis: a practical approach. Oxford: IRL Press. 1986. p. 1–36.

[50] Cardoso SM, Coimbra MA, Lopes da Silva JA. Temperature dependence of the formation and melting of pectin–Ca²⁺ networks: A rheological study. Food Hydrocolloids. 2003;17(6):801–7. doi: 10.1016/S0268-005X(03)00101-2

[51] Krämer R, Kopp F. NMR spectroscopy of vitamin C: A comprehensive study on its structure and stability in solution. Magnetic Resonance in Chemistry. 2018;56(7):593–602. doi: 10.1002/mrc.4711

[52] Hong T, Yin J-Y, Nie S-P, Xie M-Y. Applications of infrared spectroscopy in polysaccharide structural analysis: Progress, challenge and perspective. Food Chem X. 2021;12:100168. doi: 10.1016/j.fochx.2021.100168 34877528 PMC8633561

[53] Rakitikul W, Palee J. Botanical characteristics and pectin properties of Canthium parvifolium Roxb. Current Applied Science and Technology. 2018;18(3):156–66. doi: 10.14456/cast.2018.9

[54] Das K, Roy MC, Rajbanshi B, Roy MN. Assorted interactions of amino acids prevailing in aqueous vitamin C solutions probed by physicochemical and ab-initio contrivances. Chemical Physics Letters. 2017;687:209–21. doi: 10.1016/j.cplett.2017.08.054

[55] Jafari F, Behbahan SB, Azami F, Gharahgozloi M, Tadayyon B. Staff’s job satisfaction survey in Tehran’s teaching hospitals. Biomedical and Pharmacology J. 2015;7(1):09–16. doi: 10.13005/bpj/4

[56] Martins SIFS, Jongen WMF, van Boekel MAJS. A review of Maillard reaction in food and implications to kinetic modelling. Trends in Food Science & Technology. 2000;11(9–10):364–73. doi: 10.1016/s0924-2244(01)00022-x

[57] Hellwig M, Henle T. Baking, ageing, diabetes: a short history of the Maillard reaction. Angewandte Chemie International Edition. 2014;53(39):10316–29. doi: 10.1002/anie.201308808 25044982

[58] Xia T, Zhang B, Duan W, Zhang J, Wang M. Nutrients and bioactives in citrus fruits: Different citrus varieties, fruit parts, and growth seasons. Food Sci Hum Wellness. 2020;9(1):1–12. doi: 10.1016/j.fshw.2019.12.003

[59] Rotzoll N, Dunkel A, Hofmann T. Quantitative studies, taste reconstitution, and omission experiments on the key taste compounds in morel mushrooms (Morchella deliciosa Fr.). J Agric Food Chem. 2006;54(7):2705–11. doi: 10.1021/jf053131y 16569064

[60] Sandhu KS, Minhas KS. Oranges and citrus juices. In: Hui YH, editor. Handbook of fruits and fruit processing. Oxford: Blackwell Publishing. 2006. p. 309–27. doi: 10.1002/9780470277737.ch17

[61] Pegg RB, Shahidi F. Off-flavors and rancidity in foods. In: Shahidi F, editor. Handbook of food preservation. 2nd ed. Boca Raton: CRC Press. 2007. p. 55–74.

[62] Dai J, Mumper RJ. Plant phenolics: extraction, analysis and their antioxidant and anticancer properties. Molecules. 2010;15(10):7313–52. doi: 10.3390/molecules15107313 20966876 PMC6259146

[63] Prior RL, Wu X, Schaich K. Standardized methods for the determination of antioxidant capacity and phenolics in foods and dietary supplements. J Agric Food Chem. 2005;53(10):4290–302. doi: 10.1021/jf0502698 15884874

[64] Molyneux P. The use of the stable free radical diphenylpicrylhydrazyl (DPPH) for estimating antioxidant activity. Songklanakarin Journal of Science and Technology. 2004;26(2):211–9.

[65] Cai Y, Luo Q, Sun M, Corke H. Antioxidant activity and phenolic compounds of 112 traditional Chinese medicinal plants associated with anticancer. Life Sci. 2004;74(17):2157–84. doi: 10.1016/j.lfs.2003.09.047 14969719 PMC7126989

[66] Thatoi HN, Patra JK, Das SK. Free radical scavenging and antioxidant potential of mangrove plants: a review. Acta Physiol Plant. 2013;36(3):561–79. doi: 10.1007/s11738-013-1438-z

[67] Kedare SB, Singh RP. Genesis and development of DPPH method of antioxidant assay. J Food Sci Technol. 2011;48(4):412–22. doi: 10.1007/s13197-011-0251-1 23572765 PMC3551182

[68] Tournas VH, Katsoudas E. Mould and yeast flora in fresh berries, grapes and citrus fruits. Int J Food Microbiol. 2005;105(1):11–7. doi: 10.1016/j.ijfoodmicro.2005.05.002 16023239

[69] Beuchat LR. Influence of Water Activity on Growth, Metabolic Activities and Survival of Yeasts and Molds. J Food Prot. 1983;46(2):135–41. doi: 10.4315/0362-028X-46.2.135 30913613

[70] Pitt JI, Hocking AD. Fungi and food spoilage. 3rd ed. New York: Springer. 2009.

[71] Raybaudi-Massilia RM, Mosqueda-Melgar J, Soliva-Fortuny R, Martín-Belloso O. Control of Pathogenic and Spoilage Microorganisms in Fresh-cut Fruits and Fruit Juices by Traditional and Alternative Natural Antimicrobials. Compr Rev Food Sci Food Saf. 2009;8(3):157–80. doi: 10.1111/j.1541-4337.2009.00076.x 33467799

[72] Tournas V, Stack ME, Mislivec PB, Koch HA, Bandler R. Yeasts, molds and mycotoxins. FDA bacteriological analytical manual. 8th ed. Silver Spring: FDA. 2001.

