# Supplementary figures and images for: Phytochemical characterization and fungal screening of Sonneratia apetala fruit and products: Pectin and vitamin C extraction, amino acids and antioxidant activity
Source: PLoS One. 2026 Jun 26;21(6):e0352259. doi: 10.1371/journal.pone.0352259 (PMC13308825; doi:10.1371/journal.pone.0352259)

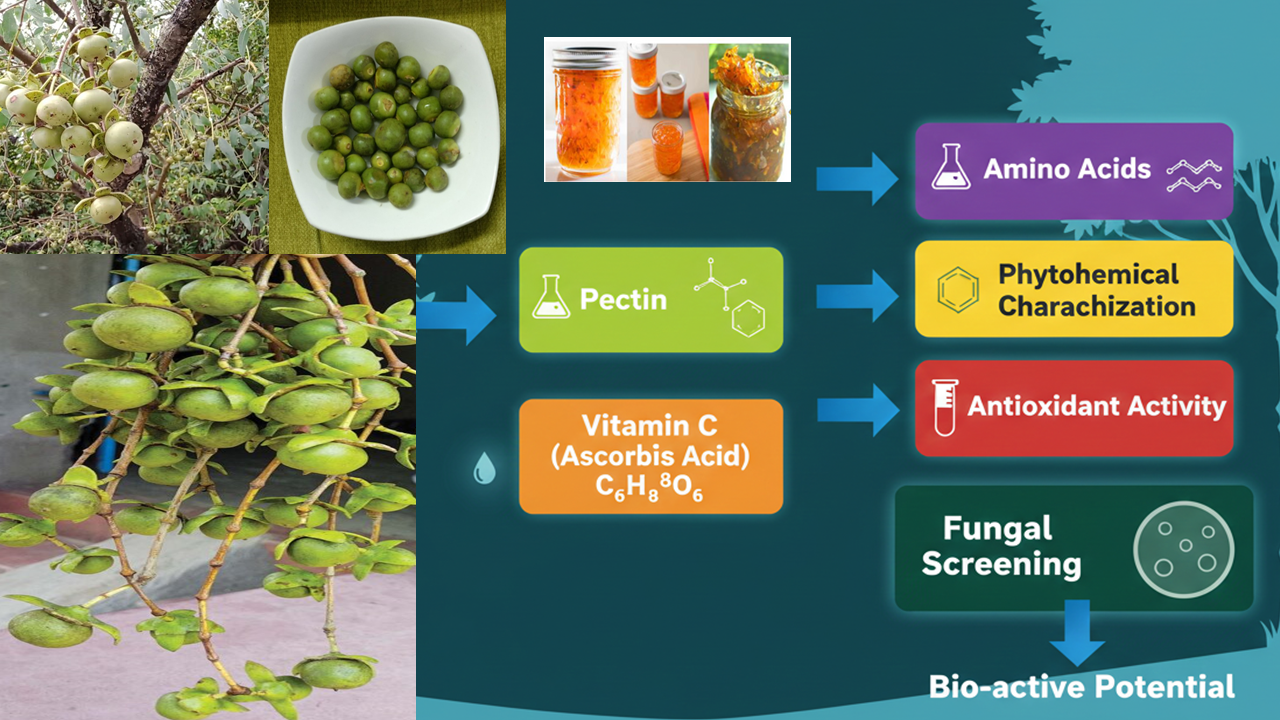

Supplement: S1 Fig — (TIF) [file pone.0352259.s001.tif]
