# Supplementary material for: Phytochemical characterization and fungal screening of Sonneratia apetala fruit and products: Pectin and vitamin C extraction, amino acids and antioxidant activity
Source: PLoS One. 2026 Jun 26;21(6):e0352259. doi: 10.1371/journal.pone.0352259 (PMC13308825; doi:10.1371/journal.pone.0352259)
Supplement: Attachment — Submitted filename: Authors response to Journal Requirment.docx [file pone.0352259.s002.docx]

We've checked your submission and before we can proceed, we need you to address the following issues:

**Q1.** Please provide a complete Data Availability Statement in the submission form, ensuring you include all necessary access information or a reason for why you are unable to make your data freely accessible. If your research concerns only data provided within your submission, please write "All data are in the manuscript and/or supporting information files" as your Data Availability Statement.

**Response:** We provide this statement in the manuscript and submission system that is “All data are in the manuscript and/or supporting information files”.

**Q2.** We note that this submission includes NMR spectroscopy data. We would recommend that you include the following information in your methods section or as Supporting Information files:

A) The make/source of the NMR instrument used in your study, as well as the magnetic field strength. For each individual experiment, please also list: the nucleus being measured; the sample concentration; the solvent in which the sample is dissolved and if solvent signal suppression was used; the reference standard and the temperature.

**Response:** We have shown it at LN 274-280.

The structures of both compounds were verified by recording 1H1H NMR spectra using a BRUKER WH 600 MHz spectrometer at BCSIR, Dhaka. For pectin analysis, samples were prepared at a concentration of 10 mg/mL in deuterated water (D₂O), while for vitamin C, a 10 mg/mL solution in deuterated dimethyl sulfoxide (DMSO-d6*d*6) was used, as CDCl₃ is unsuitable due to the poor solubility of ascorbic acid. All spectra were acquired at 298 K without the need for solvent signal suppression. Chemical shifts are reported in parts per million (ppm) relative to the tetramethylsilane (TMS; 0 ppm) reference standard [50, 51].

B) A list of the chemical shifts for all compounds characterised by NMR spectroscopy, specifying, where relevant: the chemical shift (δ), the multiplicity and the coupling constants (in Hz), for the appropriate nuclei used for assignment.

**Response:**

^1^H NMR (600 MHz, CDCl_3_): δ 3.69 (m, 1H), 3.62 (m, 5H), 3.56 (m), 3.36 (s, 1H), 2.82 (s, 92H, overlapping/solvent), 2.00 (s, 0.2H), 1.21 (s, 0.2H) for Fig 2.

^1^H NMR (600 MHz, CDCl_3_): δ 7.26 (s, solvent residual CHCl_3_), 3.35 (s, 1H), 2.88 (d, J = 34.8 Hz, 2H), 2.82 (s, overlapping signal), 2.76 (s, 1H) for Fig 6.

C) The full integrated NMR spectrum, clearly labelled with the compound name and chemical structure.

We also strongly encourage authors to provide primary NMR data files, in particular for new compounds which have not been characterised in the existing literature. Authors should provide the acquisition data, FID files and processing parameters for each experiment, clearly labelled with the compound name and identifier, as well as a structure file for each provided dataset. See our list of recommended repositories here: <https://journals.plos.org/plosone/s/recommended-repositories>.

Integrated ^1^H NMR spectra for all synthesized compounds (**4** and **8**). Each spectrum is now clearly labeled with the compound name and chemical structure. Furthermore, in accordance with the journal’s policy, we have deposited the primary NMR data including FID files, acquisition parameters, and processing scripts in the recommended repository.

**Response:** We have clearly labelled with the compound name and chemical structure in Fig. 2 and Fig.6 in the revised Manuscript. We also provide NMR data including FID files.

**Q3.** Please include a complete copy of PLOS’ questionnaire on inclusivity in global research in your revised manuscript. Our policy for research in this area aims to improve transparency in the reporting of research performed outside of researchers’ own country or community. The policy applies to researchers who have travelled to a different country to conduct research, research with Indigenous populations or their lands, and research on cultural artefacts. The questionnaire can also be requested at the journal’s discretion for any other submissions, even if these conditions are not met.  Please find more information on the policy and a link to download a blank copy of the questionnaire here: <https://journals.plos.org/plosone/s/best-practices-in-research-reporting>. Please upload a completed version of your questionnaire as Supporting Information when you resubmit your manuscript.

**Response:** We have included a complete copy of PLOS’ questionnaire on inclusivity in global research in our revised manuscript.

**Q4**. We note that the grant information you provided in the ‘Funding Information’ and ‘Financial Disclosure’ sections do not match.

When you resubmit, please ensure that you provide the correct grant numbers for the awards you received for your study in the ‘Funding Information’ section.

**Response**: We have corrected it.

**Q5**. Thank you for stating the following financial disclosure:

[The author(s) received no specific funding for this work.].

At this time, please address the following queries:

a) Please clarify the sources of funding (financial or material support) for your study. List the grants or organizations that supported your study, including funding received from your institution.

b) State what role the funders took in the study. If the funders had no role in your study, please state: “The funders had no role in study design, data collection and analysis, decision to publish, or preparation of the manuscript.”

c) If any authors received a salary from any of your funders, please state which authors and which funders.

d) If you did not receive any funding for this study, please state: “The authors received no specific funding for this work.”

Please include your amended statements within your cover letter; we will change the online submission form on your behalf.

**Response:** Please find the amended cover letter enclosed for your kind consideration.

**a) Sources of funding for this study:**

We received partial funding for this research, specifically for the purchase of the vitamin C standard only. This fund was provided by **Prof. Dr. Abubakr M. Idris** through the Deanship of Research and Graduate Studies at King Khalid University, KSA, under the Small Research Group grant (Grant No. RGP.1/266/46). This partial funding was received by the project leader, **Md. Ripaj Uddin**, during the research work through personal collaborations over the past five years. This funding was not received by our home institution.

**b) Role of the funders in the study:**

Prof. Dr. Abubakr M. Idris, who provided the partial funding, also contributed intellectually to the study. Due to initial funding limitations, the work could not be conducted properly. The project leader (Md. Ripaj Uddin) requested Prof. Idris's assistance, and together they discussed and redesigned the work. Prof. Idris supervised the revised experiments, validated the findings, provided the partial funding for the vitamin C standard, and reviewed the final manuscript. This contribution is clearly reflected in the Authors' Contribution section. No other funders had any role in study design, data collection and analysis, decision to publish, or preparation of the manuscript.

**c) Authors receiving salary from funders:**

No author received a salary from Prof. Dr. Abubakr M. Idris, King Khalid University, or any other funder for this study. The partial funding was used solely for the purchase of the vitamin C standard and did not support any author's salary or stipend.

**d) Statement regarding funding for this study:**

In the online submission form, please maintain the statement: **"The author(s) received no specific funding for this work."** This statement was originally selected because we have not received full funding for the study and, at this stage, there are no funds available for paying the Article Processing Charge (APC). We intend to apply for an APC waiver. However, to ensure full transparency, we have now provided detailed information about the partial funding (Grant No. RGP.1/266/46) in this cover letter and will also include it in the 'Funding Information' section of the manuscript, as per your instructions.

**Summary of Information for Each Query:**

| Query | Summary of Answer |
| --- | --- |
| a) Sources of funding | Partial funding from Prof. Dr. Abubakr M. Idris, King Khalid University, KSA (Grant RGP.1/266/46) for vitamin C standard only. |
| b) Role of funders | Prof. Idris contributed to study redesign, supervision, validation, and manuscript review. No other roles. |
| c) Salaries from funders | No author received a salary from any funder. |
| d) Final statement | Keep "The author(s) received no specific funding for this work" on the form for APC waiver purposes, but add detailed grant info to the manuscript. |

Q6. Thank you for your response about copyright issues with Figure 2 and Figure 6. The issue with these images is the logo for Bruker in both images. Could you please remove the logo from these images?

Response: We have removed the Bruker image logo.
