# Supplementary material for: Phytochemical characterization and fungal screening of Sonneratia apetala fruit and products: Pectin and vitamin C extraction, amino acids and antioxidant activity
Source: PLoS One. 2026 Jun 26;21(6):e0352259. doi: 10.1371/journal.pone.0352259 (PMC13308825; doi:10.1371/journal.pone.0352259)
Supplement: Peer review history

# Peer review: Decision Letter 0

**DOI:** 10.1371/journal.pone.0352259.r001

**Authors:** Hayrunnisa Nadaroglu

24 Feb 2026

PONE-D-25-66866

Phytochemical Characterization and Fungal Screening of Sonneratia apetala Fruit and Products: Pectin and Vitamin C Extraction, Amino Acids and Antioxidant Activity

PLOS One

Dear Dr. Uddin,

Thank you for submitting your manuscript to PLOS ONE. After careful consideration, we feel that it has merit but does not fully meet PLOS ONE’s publication criteria as it currently stands. Therefore, we invite you to submit a revised version of the manuscript that addresses the points raised during the review process.

Please submit your revised manuscript by Apr 10 2026 11:59PM. If you will need more time than this to complete your revisions, please reply to this message or contact the journal office at plosone@plos.org. When you're ready to submit your revision, log on to https://www.editorialmanager.com/pone/ and select the 'Submissions Needing Revision' folder to locate your manuscript file.

Please include the following items when submitting your revised manuscript:

- A letter that responds to each point raised by the academic editor and reviewer(s). You should upload this letter as a separate file labeled 'Response to Reviewers'.
- A marked-up copy of your manuscript that highlights changes made to the original version. You should upload this as a separate file labeled 'Revised Manuscript with Track Changes'.
- An unmarked version of your revised paper without tracked changes. You should upload this as a separate file labeled 'Manuscript'.

If you would like to make changes to your financial disclosure, please include your updated statement in your cover letter. Guidelines for resubmitting your figure files are available below the reviewer comments at the end of this letter.

If applicable, we recommend that you deposit your laboratory protocols in protocols.io to enhance the reproducibility of your results. Protocols.io assigns your protocol its own identifier (DOI) so that it can be cited independently in the future. For instructions see: https://journals.plos.org/plosone/s/submission-guidelines#loc-laboratory-protocols. Additionally, PLOS ONE offers an option for publishing peer-reviewed Lab Protocol articles, which describe protocols hosted on protocols.io. Read more information on sharing protocols at https://plos.org/protocols?utm_medium=editorial-email&utm_source=authorletters&utm_campaign=protocols.

We look forward to receiving your revised manuscript.

Kind regards,

Hayrunnisa Nadaroglu

Academic Editor

PLOS One

Journal Requirements:

When submitting your revision, we need you to address these additional requirements.

1. Please ensure that your manuscript meets PLOS ONE's style requirements, including those for file naming. The PLOS ONE style templates can be found at

https://journals.plos.org/plosone/s/file?id=wjVg/PLOSOne_formatting_sample_main_body.pdf and https://journals.plos.org/plosone/s/file?id=ba62/PLOSOne_formatting_sample_title_authors_affiliations.pdf

2. In your Methods section, please provide additional information regarding the permits you obtained for the work. Please ensure you have included the full name of the authority that approved the field site access and, if no permits were required, a brief statement explaining why.

3. We note that this submission includes NMR spectroscopy data. We would recommend that you include the following information in your methods section or as Supporting Information files:

1) The make/source of the NMR instrument used in your study, as well as the magnetic field strength. For each individual experiment, please also list: the nucleus being measured; the sample concentration; the solvent in which the sample is dissolved and if solvent signal suppression was used; the reference standard and the temperature.

2) A list of the chemical shifts for all compounds characterised by NMR spectroscopy, specifying, where relevant: the chemical shift (δ), the multiplicity and the coupling constants (in Hz), for the appropriate nuclei used for assignment.

3)The full integrated NMR spectrum, clearly labelled with the compound name and chemical structure.

We also strongly encourage authors to provide primary NMR data files, in particular for new compounds which have not been characterised in the existing literature. Authors should provide the acquisition data, FID files and processing parameters for each experiment, clearly labelled with the compound name and identifier, as well as a structure file for each provided dataset. See our list of recommended repositories here: https://journals.plos.org/plosone/s/recommended-repositories

4. Please include a complete copy of PLOS’ questionnaire on inclusivity in global research in your revised manuscript. Our policy for research in this area aims to improve transparency in the reporting of research performed outside of researchers’ own country or community. The policy applies to researchers who have travelled to a different country to conduct research, research with Indigenous populations or their lands, and research on cultural artefacts. The questionnaire can also be requested at the journal’s discretion for any other submissions, even if these conditions are not met.  Please find more information on the policy and a link to download a blank copy of the questionnaire here: https://journals.plos.org/plosone/s/best-practices-in-research-reporting. Please upload a completed version of your questionnaire as Supporting Information when you resubmit your manuscript.

5. We note that the grant information you provided in the ‘Funding Information’ and ‘Financial Disclosure’ sections do not match.

When you resubmit, please ensure that you provide the correct grant numbers for the awards you received for your study in the ‘Funding Information’ section.

6. Thank you for stating the following financial disclosure:

[The author(s) received no specific funding for this work.].

At this time, please address the following queries:

a) Please clarify the sources of funding (financial or material support) for your study. List the grants or organizations that supported your study, including funding received from your institution.

b) State what role the funders took in the study. If the funders had no role in your study, please state: “The funders had no role in study design, data collection and analysis, decision to publish, or preparation of the manuscript.”

c) If any authors received a salary from any of your funders, please state which authors and which funders.

d) If you did not receive any funding for this study, please state: “The authors received no specific funding for this work.”

Please include your amended statements within your cover letter; we will change the online submission form on your behalf.

7. Thank you for stating the following in your manuscript:

[King Khalid University’s Deanship of Scientific Research for funding via grant RGP 2/157/46.]

We note that you have provided funding information that is not currently declared in your Funding Statement. However, funding information should not appear in the Acknowledgments section or other areas of your manuscript. We will only publish funding information present in the Funding Statement section of the online submission form.

Please remove any funding-related text from the manuscript and let us know how you would like to update your Funding Statement. Currently, your Funding Statement reads as follows:

[The author(s) received no specific funding for this work.]

Please include your amended statements within your cover letter; we will change the online submission form on your behalf.

8. Please provide a complete Data Availability Statement in the submission form, ensuring you include all necessary access information or a reason for why you are unable to make your data freely accessible. If your research concerns only data provided within your submission, please write "All data are in the manuscript and/or supporting information files" as your Data Availability Statement.

9. We note that Figures 2 and 6 in your submission contain copyrighted images. All PLOS content is published under the Creative Commons Attribution License (CC BY 4.0), which means that the manuscript, images, and Supporting Information files will be freely available online, and any third party is permitted to access, download, copy, distribute, and use these materials in any way, even commercially, with proper attribution. For more information, see our copyright guidelines: http://journals.plos.org/plosone/s/licenses-and-copyright.

We require you to either (1) present written permission from the copyright holder to publish these figures specifically under the CC BY 4.0 license, or (2) remove the figures from your submission:

1. You may seek permission from the original copyright holder of Figure 2 and 6 to publish the content specifically under the CC BY 4.0 license.

We recommend that you contact the original copyright holder with the Content Permission Form (http://journals.plos.org/plosone/s/file?id=7c09/content-permission-form.pdf) and the following text:

“I request permission for the open-access journal PLOS ONE to publish XXX under the Creative Commons Attribution License (CCAL) CC BY 4.0 (http://creativecommons.org/licenses/by/4.0/). Please be aware that this license allows unrestricted use and distribution, even commercially, by third parties. Please reply and provide explicit written permission to publish XXX under a CC BY license and complete the attached form.”

Please upload the completed Content Permission Form or other proof of granted permissions as an "Other" file with your submission.

In the figure caption of the copyrighted figure, please include the following text: “Reprinted from [ref] under a CC BY license, with permission from [name of publisher], original copyright [original copyright year].”

2. If you are unable to obtain permission from the original copyright holder to publish these figures under the CC BY 4.0 license or if the copyright holder’s requirements are incompatible with the CC BY 4.0 license, please either i) remove the figure or ii) supply a replacement figure that complies with the CC BY 4.0 license. Please check copyright information on all replacement figures and update the figure caption with source information. If applicable, please specify in the figure caption text when a figure is similar but not identical to the original image and is therefore for illustrative purposes only.

If the reviewer comments include a recommendation to cite specific previously published works, please review and evaluate these publications to determine whether they are relevant and should be cited. There is no requirement to cite these works unless the editor has indicated otherwise.

[Note: HTML markup is below. Please do not edit.]

Reviewers' comments:

Reviewer's Responses to Questions

**Comments to the Author** 

1. Is the manuscript technically sound, and do the data support the conclusions?

The manuscript must describe a technically sound piece of scientific research with data that supports the conclusions. Experiments must have been conducted rigorously, with appropriate controls, replication, and sample sizes. The conclusions must be drawn appropriately based on the data presented.

Reviewer #1: Partly

Reviewer #2: Yes

Reviewer #3: Yes

**********

2. Has the statistical analysis been performed appropriately and rigorously?

Reviewer #1: No

Reviewer #2: Yes

Reviewer #3: Yes

**********

3. Have the authors made all data underlying the findings in their manuscript fully available?

The PLOS Data policy requires authors to make all data underlying the findings described in their manuscript fully available without restriction, with rare exception (please refer to the Data Availability Statement in the manuscript PDF file). The data should be provided as part of the manuscript or its supporting information, or deposited to a public repository. For example, in addition to summary statistics, the data points behind means, medians and variance measures should be available. If there are restrictions on publicly sharing data—e.g. participant privacy or use of data from a third party—those must be specified.

Reviewer #1: Yes

Reviewer #2: Yes

Reviewer #3: Yes

**********

4. Is the manuscript presented in an intelligible fashion and written in standard English?

PLOS ONE does not copyedit accepted manuscripts, so the language in submitted articles must be clear, correct, and unambiguous. Any typographical or grammatical errors should be corrected at revision, so please note any specific errors here.

Reviewer #1: No

Reviewer #2: Yes

Reviewer #3: No

**********

5. Review Comments to the Author

Please use the space provided to explain your answers to the questions above. You may also include additional comments for the author, including concerns about dual publication, research ethics, or publication ethics. (Please upload your review as an attachment if it exceeds 20,000 characters)

Reviewer #1: The authors present a comprehensive analytical investigation of Sonneratia apetala fruit and its processed products, addressing phytochemical composition, nutritional parameters, antioxidant capacity, and microbial safety. The study is ambitious in scope and makes use of established analytical techniques. The topic is timely and relevant, particularly in the context of underutilized natural resources and food innovation.

The experimental approaches employed are generally appropriate for the stated objectives, and the dataset is substantial. However, several aspects of methodological reporting and data interpretation require clarification. In particular, details regarding experimental replication, statistical assumptions, and derivation of key parameters (such as IC₅₀ values) should be more clearly presented to allow full assessment of analytical rigor.

Some conclusions extend beyond what can be directly supported by the data. Claims related to nutraceutical application, health impact, and industrial scalability would benefit from a more cautious framing as future perspectives rather than definitive outcomes. Similarly, the absence of fungal growth in processed products should be interpreted primarily as evidence of microbiological safety and effective processing, rather than intrinsic antifungal activity.

The reported antioxidant activity, especially for the ethyl acetate root extract, is noteworthy and merits further investigation. At the same time, comparisons with synthetic antioxidants should be confined to in vitro chemical assays, without implying biological or therapeutic equivalence.

The manuscript is generally understandable, but it would benefit from careful language editing. There are frequent grammatical and stylistic issues, as well as some redundancy, which should be addressed during revision. Clear, concise language will substantially improve readability and impact, particularly as the journal does not provide copyediting.

Overall, this work has the potential to make a meaningful contribution to the field. With revisions focused on clarifying methods, moderating claims, strengthening statistical transparency, and improving language quality, the manuscript could be significantly improved and better aligned with the journal’s publication standards.

Reviewer #2: Phytochemical Characterization and Fungal Screening of Sonneratia apetala Fruit and

Products: Pectin and Vitamin C Extraction, Amino Acids and Antioxidant Activity

Major corrections to the article titled are listed below;

1-Literature should be added to line 104 “Preparation of Crude Extracts”.

2-The brands of the chemicals used in the study should be listed.

3-The location where the work was carried out and any services procured should be specified.

4- The plant name Sonneratia apetala is written in italics in some places and in plain text in others. It should be written in standard italics. The entire article should be reviewed. For example, line 424.

5-If services were procured for characterization studies, this should be indicated.

6-Reference number 1, line 548, is missing.

7-All reference writing rules should be checked. Also, the numbering of references in the article should be checked. For example, reference 42 on line 218 is incorrect.

8-Literature should be added to line 225.

9-In the references, the volume number is written plain in some literature and italic in others. Corrections should be made according to spelling rules.

10-In the references, some literature has its DOI number underlined, while others do not. For example, line 628 and line 605.

11- Line 481, section 3.4: Antifungal activity determination results should be presented in tabular form. Reference microorganism names should be given.

12-In the table, line 433, which is expressed as EA Sa Root, is expressed as ES sA root. This needs to be corrected.

13-Table 2 shows that comparisons of antioxidant results with positive controls such as BHA and BHT should be made relative to the positive control.,

14-All graphs in the article, from Figure 1 to Figure 8, should be redrawn based on the raw data. They should be clear and understandable.

15- Article spelling and grammar rules should be reviewed.

Reviewer #3: In this study, the authors aimed to investigate a comprehensive biochemical and microbiological profile of S. apetala fruit and its derived products (jam, jelly, and pickle), integrating nutritional valorization with safety assessment..

Overall, the manuscript is of interest to the readership. However, some aspects need to be considered and revised.

Introduction

- Please split the first paragraph into two separate paragraphs.

- Please add references to “Although some nutritional potential of mangrove species has been previously reported,...”

- Please revise the sentence “Assessment of in vitro antioxidant activity by DPPH assay.”

- Please add the main objective of this study to the last paragraph.

- I think that “Quantitatively, pectin can be determined by high-performance liquid chromatography for the most common carbohydrates and gene-dataistic analysis to measure vitamin C and amino acids structure elucidation and confirmation for FTIR (Fourier-transform infrared spectrometer) spectroscopy as well as NMR (nuclear magnetic resonance) spectroscopy.” is not relevant for the introduction. Please remove it and add my first suggestion.

Methods

- “Nijumdwip, Hatiya”, please add the country.

- gm should be written as g (or mg), please correct it.

- “The TPC was determined spectrophotometrically using the method” should be “TPC was determined using a spectrophotometric method”.

- Why did the authors add section “2.7.2. HPLC Analysis”?

- Please define the study groups for statistical analysis.

Results and Discussion

- Please discuss in the section Comparative Analysis of Amino Acid Profiles the nutritional value (in aspects EAA) according to the author's statement “Thus, profiling EAA composition of S. apetala fruit might reveal not only its nutritional potential, but also suggests remedies for the protein malnutrition in underdeveloped countries.”

- As my suggestion above, please add the nutritional aspect to other sections. Because the authors begin the article by stating, "The rising world demand for sustainable and natural bioresource has rekindled interest in underexploited plants with promising nutritional and industrial applications".

- Authors should add a Strengths and Limitations section.

**********

6. PLOS authors have the option to publish the peer review history of their article (what does this mean?). If published, this will include your full peer review and any attached files.

If you choose “no”, your identity will remain anonymous but your review may still be made public.

**Do you want your identity to be public for this peer review?**    For information about this choice, including consent withdrawal, please see our Privacy Policy.

Reviewer #1: **Yes:**    Ashraful Hoque

Reviewer #2: No

Reviewer #3: No

**********

[NOTE: If reviewer comments were submitted as an attachment file, they will be attached to this email and accessible via the submission site. Please log into your account, locate the manuscript record, and check for the action link "View Attachments". If this link does not appear, there are no attachment files.]

To ensure your figures meet our technical requirements, please review our figure guidelines: https://journals.plos.org/plosone/s/figures

You may also use PLOS’s free figure tool, NAAS, to help you prepare publication quality figures: https://journals.plos.org/plosone/s/figures#loc-tools-for-figure-preparation.

NAAS will assess whether your figures meet our technical requirements by comparing each figure against our figure specifications.

# Author comment: Author response to Decision Letter 1

**DOI:** 10.1371/journal.pone.0352259.r002

8 Apr 2026

The

Editor in Chief

PLOS ONE

Subject: Submission of review comments of the manuscript: “PONE-D-25-66866”

Dear Editor

I am submitting the review comments of the manuscript “Phytochemical Characterization and Fungal Screening of Sonneratia apetala Fruit and Products: Pectin and Vitamin C Extraction, Amino Acids and Antioxidant Activity”. The authors would like to start by thanking the esteemed reviewers for their thoughtful comments and suggestions. A few points have been raised for clarification and further improvement of the paper. We have addressed each comment/suggestion made by the reviewers one by one below. We have also mentioned the changes made in the revised manuscript (m/s) below. All the changes in the revised m/s have been highlighted in Yellow and track change format.

Reviewer #1:

Q1: The authors present a comprehensive analytical investigation of Sonneratia apetala fruit and its processed products, addressing phytochemical composition, nutritional parameters, antioxidant capacity, and microbial safety. The study is ambitious in scope and makes use of established analytical techniques. The topic is timely and relevant, particularly in the context of underutilized natural resources and food innovation.

Response: Thank you for your positive feedback.

Q2: The experimental approaches employed are generally appropriate for the stated objectives, and the data set is substantial. However, several aspects of methodological reporting and data interpretation require clarification. In particular, details regarding experimental replication, statistical assumptions, and derivation of key parameters (such as IC₅₀ values) should be more clearly presented to allow full assessment of analytical rigor.

Response: This has been explained in lines 454–475 of the revised manuscript.

Q3: Some conclusions extend beyond what can be directly supported by the data. Claims related to nutraceutical application, health impact, and industrial scalability would benefit from a more cautious framing as future perspectives rather than definitive outcomes. Similarly, the absence of fungal growth in processed products should be interpreted primarily as evidence of microbiological safety and effective processing, rather than intrinsic antifungal activity.

Response: This has been corrected at subsection 3.4.

Q4: The reported antioxidant activity, especially for the ethyl acetate root extract, is noteworthy and merits further investigation. At the same time, comparisons with synthetic antioxidants should be confined to in vitro chemical assays, without implying biological or therapeutic equivalence.

Response: This has been corrected in Table 2.

Q5: The manuscript is generally understandable, but it would benefit from careful language editing. There are frequent grammatical and stylistic issues, as well as some redundancy, which should be addressed during revision. Clear, concise language will substantially improve readability and impact, particularly as the journal does not provide copyediting.

Response: Grammatical and stylistic issues have been addressed in the revised manuscript.

Q6: Overall, this work has the potential to make a meaningful contribution to the field. With revisions focused on clarifying methods, moderating claims, strengthening statistical transparency, and improving language quality, the manuscript could be significantly improved and better aligned with the journal’s publication standards.

Response: We have corrected it according to your suggestions.

Reviewer #2:

Phytochemical Characterization and Fungal Screening of Sonneratia apetala Fruit and

Products: Pectin and Vitamin C Extraction, Amino Acids and Antioxidant Activity

Major Corrections to the article titled are listed below;

Q1-Literature should be added to line 104 “Preparation of Crude Extracts”.

Response: We have cited this part in the revised manuscript.

de Carvalho, A. T., Paes, M. M., Cunha, M. S., Brandão, G. C., Mapeli, A. M., Rescia, V. C., ... & Villas-Boas, G. R. (2020). Ethnopharmacology of fruit plants: a literature review on the toxicological, phytochemical, cultural aspects, and a mechanistic approach to the pharmacological effects of four widely used species. Molecules, 25(17), 3879. https://doi.org/10.3390/molecules25173879

Q2-The brands of the chemicals used in the study should be listed.

Response: The chemical names and brands have been incorporated at lines 113–119 of the revised manuscript.

Q3-The location where the work was carried out and any services procured should be specified.

Response: We have incorporated it at lines 103–104 in the revised manuscript.

Q4- The plant name Sonneratia apetala is written in italics in some places and in plain text in others. It should be written in standard italics. The entire article should be reviewed. For example, line 424.

Response: We have corrected it in the revised version manuscript.

Q5-If services were procured for characterization studies, this should be indicated.

Response: We have mention it in the revised manuscript.

Q6-Reference number 1, line 548, is missing.

Response: The relevant reference has been cited.

Q7-All reference writing rules should be checked. Also, the numbering of references in the article should be checked. For example, reference 42 on line 218 is incorrect.

Response: We have corrected it in the revised version manuscript.

Q8-Literature should be added to line 225.

Response: The relevant reference has been cited.

Q9-In the references, the volume number is written plain in some literature and italic in others. Corrections should be made according to spelling rules.

Response: We have corrected all reference in the same format.

Q10-In the references, some literature has its DOI number underlined, while others do not. For example, line 628 and line 605.

Response: The necessary correction has been made.

Q11- Line 481, section 3.4: Antifungal activity determination results should be presented in tabular form. Reference microorganism names should be given.

Response: The relevant information has been added to Table 3.

Q12-In the table, line 433, which is expressed as EA Sa Root, is expressed as ES sA root. This needs to be corrected.

Response: This has been corrected.

Q13-Table 2 shows that comparisons of antioxidant results with positive controls such as BHA and BHT should be made relative to the positive control.

Response: The BHA and BHT results have been incorporated and presented relative to the positive control.

Q14-All graphs in the article, from Figure 1 to Figure 8, should be redrawn based on the raw data. They should be clear and understandable.

Response: All figures have been redrawn using the raw data.

Q15- Article spelling and grammar rules should be reviewed.

Response: We have thoroughly reviewed the whole manuscript.

Reviewer #3:

In this study, the authors aimed to investigate a comprehensive biochemical and microbiological profile of S. apetala fruit and its derived products (jam, jelly, and pickle), integrating nutritional valorization with safety assessment.nOverall, the manuscript is of interest to the readership. However, some aspects need to be considered and revised.

Introduction

Q1- Please split the first paragraph into two separate paragraphs.

Response: We have split the first paragraph into two separate paragraphs.

Q2- Please add references to “Although some nutritional potential of mangrove species has been previously reported,...”

Response: As requested, we have added this citation at line 71.

Q3- Please revise the sentence “Assessment of in vitro antioxidant activity by DPPH assay.”

Response: As requested, we have revised this sentence at line 75.

Q4- Please add the main objective of this study to the last paragraph.

Response: We have now corrected this issue.

Q5- I think that “Quantitatively, pectin can be determined by high-performance liquid chromatography for the most common carbohydrates and gene-dataistic analysis to measure vitamin C and amino acids structure elucidation and confirmation for FTIR (Fourier-transform infrared spectrometer) spectroscopy as well as NMR (nuclear magnetic resonance) spectroscopy.” is not relevant for the introduction. Please remove it and add my first suggestion.

Response: The text has been removed as requested.

Methods

Q6- “Nijumdwip, Hatiya”, please add the country.

Response: The requested information has been added.

Q7- gm should be written as g (or mg), please correct it.

Response: We have written it correctly.

Q8- “The TPC was determined spectrophotometrically using the method” should be “TPC was determined using a spectrophotometric method”.

Response: The necessary correction has been made.

Q9- Why did the authors add section “2.7.2. HPLC Analysis”?

Response: The necessary correction has been made.

Q10- Please define the study groups for statistical analysis.

Response: The requested information has been incorporated at lines 280–294.

Results and Discussion

Q11- Please discuss in the section Comparative Analysis of Amino Acid Profiles the nutritional value (in aspects EAA) according to the author's statement “Thus, profiling EAA composition of S. apetala fruit might reveal not only its nutritional potential, but also suggests remedies for the protein malnutrition in underdeveloped countries.”

Response: This sentence has been corrected in lines 64–66 of the revised manuscript.

Q12- As my suggestion above, please add the nutritional aspect to other sections. Because the authors begin the article by stating, "The rising world demand for sustainable and natural bioresource has rekindled interest in underexploited plants with promising nutritional and industrial applications".

Response: The requested information has been incorporated at lines 540–568.

Q13- Authors should add a Strengths and Limitations section.

Response: The requested information has been incorporated at lines 540–568.

Attachment Submitted filename: Authors response to Journal Requirment.docx

# Peer review: Decision Letter 1

**DOI:** 10.1371/journal.pone.0352259.r003

**Authors:** Hayrunnisa Nadaroglu, Hayrunnisa Nadaroglu

9 Jun 2026

Phytochemical Characterization and Fungal Screening of Sonneratia apetala Fruit and Products: Pectin and Vitamin C Extraction, Amino Acids and Antioxidant Activity

PONE-D-25-66866R1

Dear Dr. Uddin,

We’re pleased to inform you that your manuscript has been judged scientifically suitable for publication and will be formally accepted for publication once it meets all outstanding technical requirements.

Within one week, you’ll receive an e-mail detailing the required amendments. When these have been addressed, you’ll receive a formal acceptance letter and your manuscript will be scheduled for publication.

An invoice will be generated when your article is formally accepted. Please note, if your institution has a publishing partnership with PLOS and your article meets the relevant criteria, all or part of your publication costs will be covered. Please make sure your user information is up-to-date by logging into Editorial Manager at Editorial Manager® and clicking the ‘Update My Information' link at the top of the page. For questions related to billing, please contact billing support.

If your institution or institutions have a press office, please notify them about your upcoming paper to help maximize its impact. If they’ll be preparing press materials, please inform our press team as soon as possible -- no later than 48 hours after receiving the formal acceptance. Your manuscript will remain under strict press embargo until 2 pm Eastern Time on the date of publication. For more information, please contact onepress@plos.org.

Kind regards,

Hayrunnisa Nadaroglu

Academic Editor

PLOS One

Additional Editor Comments (optional):

Reviewers' comments:

Reviewer's Responses to Questions

**Comments to the Author** 

1. If the authors have adequately addressed your comments raised in a previous round of review and you feel that this manuscript is now acceptable for publication, you may indicate that here to bypass the “Comments to the Author” section, enter your conflict of interest statement in the “Confidential to Editor” section, and submit your "Accept" recommendation.

Reviewer #1: All comments have been addressed

Reviewer #2: All comments have been addressed

Reviewer #3: All comments have been addressed

**********

2. Is the manuscript technically sound, and do the data support the conclusions?

The manuscript must describe a technically sound piece of scientific research with data that supports the conclusions. Experiments must have been conducted rigorously, with appropriate controls, replication, and sample sizes. The conclusions must be drawn appropriately based on the data presented.

Reviewer #1: Yes

Reviewer #2: Yes

Reviewer #3: Yes

**********

3. Has the statistical analysis been performed appropriately and rigorously?

Reviewer #1: Yes

Reviewer #2: Yes

Reviewer #3: Yes

**********

4. Have the authors made all data underlying the findings in their manuscript fully available?

The PLOS Data policy requires authors to make all data underlying the findings described in their manuscript fully available without restriction, with rare exception (please refer to the Data Availability Statement in the manuscript PDF file). The data should be provided as part of the manuscript or its supporting information, or deposited to a public repository. For example, in addition to summary statistics, the data points behind means, medians and variance measures should be available. If there are restrictions on publicly sharing data—e.g. participant privacy or use of data from a third party—those must be specified.

Reviewer #1: Yes

Reviewer #2: Yes

Reviewer #3: Yes

**********

5. Is the manuscript presented in an intelligible fashion and written in standard English?

PLOS ONE does not copyedit accepted manuscripts, so the language in submitted articles must be clear, correct, and unambiguous. Any typographical or grammatical errors should be corrected at revision, so please note any specific errors here.

Reviewer #1: Yes

Reviewer #2: Yes

Reviewer #3: Yes

**********

6. Review Comments to the Author

Please use the space provided to explain your answers to the questions above. You may also include additional comments for the author, including concerns about dual publication, research ethics, or publication ethics. (Please upload your review as an attachment if it exceeds 20,000 characters)

Reviewer #1: (No Response)

Reviewer #2: The author appears to have completed all the missing information given in the first revision. Çalışmanın yayınlanmasını kabul ediyorum

Reviewer #3: Thank you for the opportunity to review the revised manuscript entitled “Phytochemical Characterization and Fungal Screening of Sonneratia apetala Fruit and Products: Pectin and Vitamin C Extraction, Amino Acids and Antioxidant Activity”. In the revised manuscript, the authors revised many of the recommendations. I have no more suggestions.

**********

7. PLOS authors have the option to publish the peer review history of their article (what does this mean?). If published, this will include your full peer review and any attached files.

If you choose “no”, your identity will remain anonymous but your review may still be made public.

**Do you want your identity to be public for this peer review?**    For information about this choice, including consent withdrawal, please see our Privacy Policy.

Reviewer #1: **Yes:**    Ashraful Hoque

Reviewer #2: No

Reviewer #3: No

**********

# Editor report: Acceptance letter

**DOI:** 10.1371/journal.pone.0352259.r004

**Authors:** Hayrunnisa Nadaroglu, Hayrunnisa Nadaroglu

PONE-D-25-66866R1

PLOS One

Dear Dr. Uddin,

I'm pleased to inform you that your manuscript has been deemed suitable for publication in PLOS One. Congratulations! Your manuscript is now being handed over to our production team.

At this stage, our production department will prepare your paper for publication. This includes ensuring the following:

* All references, tables, and figures are properly cited

* All relevant supporting information is included in the manuscript submission,

* There are no issues that prevent the paper from being properly typeset

You will receive further instructions from the production team, including instructions on how to review your proof when it is ready. Please keep in mind that we are working through a large volume of accepted articles, so please give us a few days to review your paper and let you know the next and final steps.

Lastly, if your institution or institutions have a press office, please let them know about your upcoming paper now to help maximize its impact. If they'll be preparing press materials, please inform our press team within the next 48 hours. Your manuscript will remain under strict press embargo until 2 pm Eastern Time on the date of publication. For more information, please contact onepress@plos.org.

You will receive an invoice from PLOS for your publication fee after your manuscript has reached the completed accept phase. If you receive an email requesting payment before acceptance or for any other service, this may be a phishing scheme. Learn how to identify phishing emails and protect your accounts at https://explore.plos.org/phishing.

If we can help with anything else, please email us at customercare@plos.org.

Thank you for submitting your work to PLOS ONE and supporting open access.

Kind regards,

PLOS ONE Editorial Office Staff

on behalf of

Professor Hayrunnisa Nadaroglu

Academic Editor

PLOS One
